# Ibandronate Use in Osteoporotic Vertebral Fractures: A Retrospective Clinical Study Integrated with Exploratory Network Pharmacology and Cross-Cohort Transcriptomic Analysis

**DOI:** 10.3390/biomedicines14061315

**Published:** 2026-06-10

**Authors:** Mehmet Albayrak, Ersin Guner, Fatih Ugur, Ibrahim Yilmaz

**Affiliations:** 1 Department of Medical Services and Techniques, Vocational School of Health Services, Istanbul Rumeli University, Istanbul 34570, Turkey; 2 Department of Pharmacy, Konya Numune Hospital, Ministry of Health of the Republic of Turkey, Konya 42060, Turkey; 3 Department of Orthopedics and Traumatology, Kastamonu University Faculty of Medicine, Kastamonu 37200, Turkey; 4 Unit of Pharmacovigilance, Dr. Ismail Fehmi Cumalioglu City Hospital, Ministry of Health of the Republic of Turkey, Tekirdag 59020, Turkey

**Keywords:** ibandronate, osteoporosis, vertebral fracture, pain, network pharmacology, transcriptomics

## Abstract

**Background:** Ibandronate is a nitrogen-containing bisphosphonate used in osteoporosis; however, its relationship with vertebral-fracture-related outcomes, pain trajectories, and broader inflammatory–skeletal signaling remains incompletely characterized. **Methods:** This retrospective observational study included patients with osteoporosis categorized according to ibandronate exposure. The primary outcome was new vertebral fracture occurrence, and the secondary outcome was change in pain severity assessed using the Visual Analog Scale (VAS). Multivariable regression, sensitivity analyses, and exploratory network-pharmacology, transcriptomic, and molecular docking analyses were performed. **Results:** Forty patients (20 ibandronate, 20 control) were included. Ibandronate use was associated with numerically lower vertebral fracture occurrence, although this did not reach statistical significance in crude or adjusted analyses. Greater pain reduction was observed in unadjusted analyses but was attenuated after multivariable adjustment, and baseline heterogeneity should be considered when interpreting between-group differences. Radiological outcomes did not differ significantly between groups. Exploratory systems-level analyses identified enrichment patterns involving inflammatory signaling, osteoclast differentiation, cytokine-associated pathways, and skeletal regulatory processes; however, these findings should be interpreted as hypothesis-generating and not as evidence of causal biological mechanisms. **Conclusions:** In this exploratory, hypothesis-generating study, ibandronate use was associated with trends toward lower vertebral fracture occurrence and greater unadjusted pain improvement, although these findings were attenuated after adjustment. The combined clinical, transcriptomic, and computational observations are compatible with the possibility that inflammatory and skeletal regulatory pathways may intersect within a broader systems-level framework relevant to vertebral-fracture-related outcomes in osteoporosis. However, these findings should not be interpreted as direct mechanistic evidence of ibandronate-specific molecular activity or clinical efficacy. Larger prospective studies integrating clinical, radiological, and mechanistic approaches are required to clarify the biological and clinical relevance of these observations.

## 1. Introduction

Osteoporosis is a chronic skeletal disorder characterized by reduced bone strength and increased susceptibility to fragility fractures, with vertebral fractures representing one of its most frequent and clinically consequential manifestations [1,2]. Osteoporotic vertebral fractures are associated not only with structural collapse and future fracture risk, but also with persistent pain, functional limitation, impaired mobility, and deterioration in quality of life [1]. Recent clinical reviews and treatment updates have continued to emphasize that fracture prevention remains the central goal of osteoporosis management, yet symptomatic outcomes, particularly pain, are often less clearly resolved than radiographic or densitometric endpoints [2,3].

Bisphosphonates remain a cornerstone of antiresorptive therapy in osteoporosis, and ibandronate continues to be used in routine practice because of its established antiresorptive profile and practical dosing schedule [2,3,4]. Mechanistically, nitrogen-containing bisphosphonates inhibit farnesyl pyrophosphate synthase (FDPS) within the mevalonate pathway, thereby suppressing osteoclast function and bone resorption [3]. At the same time, recent evidence suggests that the clinical effects of antiresorptive agents should not be interpreted solely through changes in bone mineral density, because fracture outcomes, treatment sequencing, and real-world treatment responses remain heterogeneous across patient populations [2,3]. In this context, a recent individual patient-level meta-analysis of placebo-controlled bisphosphonate trials, based on the available ibandronate datasets, reported favorable effects on bone mineral density and overall fracture risk while also emphasizing the need for clinically cautious interpretation across different populations and treatment settings [4].

From a pharmacological perspective, pain in osteoporotic vertebral fractures is unlikely to reflect a purely structural phenomenon. Increasing evidence supports a biologically relevant interface between bone remodeling and inflammatory signaling, in which osteoclast activity, immune mediators, and cytokine-driven pathways may jointly shape skeletal deterioration and symptom generation [5]. Age-related low-grade inflammation has been implicated in the pathophysiology of bone loss, osteoclastogenesis, and impaired skeletal homeostasis, supporting the broader concept that inflammatory biology may influence not only fracture susceptibility but also pain-related manifestations of osteoporosis [5]. This framework is particularly relevant in situations where radiological progression and symptomatic trajectories may not necessarily occur in parallel. Despite the widespread clinical use of ibandronate, data simultaneously evaluating vertebral fracture outcomes, pain trajectories, and radiological evolution in real-world osteoporosis cohorts remain limited. Furthermore, the extent to which clinical observations can be interpreted within a broader systems-level biological framework integrating bone remodeling, inflammatory signaling, and pain-related pathways has not been extensively explored.

On this basis, we considered it insufficient to examine ibandronate only as an antiresorptive agent linked to a single canonical target. Instead, we approached the problem as a network-level pharmacological question involving bone remodeling, inflammatory signaling, and nociceptive biology. Recent osteoporosis studies have increasingly used integrative network pharmacology to move beyond one-target/one-pathway models, combining protein–protein interaction (PPI) mapping, topological prioritization, enrichment analysis, and molecular docking to identify biologically coherent target clusters and candidate mechanisms [6,7]. Within such a framework, PPI analysis is informative for defining how curated targets interact as a system, Cytoscape-based topology analysis helps prioritize structurally central nodes within that system, disease enrichment analysis helps determine whether the selected target architecture maps back to clinically meaningful pathological phenotypes, and molecular docking provides a complementary means of examining the plausibility of ligand–target interactions at the molecular level [6,7]. In the present study, these analyses were not included as decorative add-ons; rather, they were used to test whether the clinical signal observed around ibandronate could be interpreted within a biologically coherent network linking osteoclast regulation, inflammatory mediators, and pain-related pathways [6,7].

We therefore hypothesized that ibandronate-associated clinical effects in patients with vertebral fractures might extend beyond a narrowly structural antiresorptive effect and instead be embedded within a broader interaction network connecting osteoclast-centered bone resorption, inflammatory signaling, and pain-related molecular pathways [5,6,7]. Under this hypothesis, a systems-level analytical strategy was considered particularly appropriate, because it permits simultaneous evaluation of target connectivity, functional clustering, disease relevance, and ligand–protein plausibility rather than forcing interpretation through a single mechanistic axis [5,6,7].

Accordingly, the aim of this study was to evaluate the association of ibandronate use with vertebral-fracture-related clinical outcomes in an osteoporosis cohort, with particular attention to new vertebral fractures, pain trajectories, and radiological evolution. To strengthen the biological context of these clinical observations, the study further integrated exploratory network pharmacology, molecular docking, and cross-cohort transcriptomic analyses using publicly available GEO datasets. This integrative strategy was intended to examine whether the clinical and computational signals converged around bone remodeling, inflammatory signaling, and pain-related molecular pathways. Given the retrospective clinical design, small sample size, and inferential nature of the in silico and transcriptomic analyses, the present work was conceived as exploratory and hypothesis-generating rather than confirmatory.

## 2. Materials and Methods

### 2.1. Study Design and Ethical Approval

This single-center retrospective observational study was conducted in a tertiary care setting. The reporting of the study was aligned with the Strengthening the Reporting of Observational Studies in Epidemiology (STROBE) statement [8]. Ethical approval was obtained from the Istanbul Rumeli University Ethics Committee (Meeting No: 2026/04; Decision No: 15; Date: 29 April 2026). All procedures were carried out in accordance with the ethical standards of the institutional and national research committees, as well as the Declaration of Helsinki (2013 revision). Given the retrospective design and the use of anonymized clinical data, the requirement for written informed consent was waived by the ethics committee.

### 2.2. Study Population

Patients with a diagnosis of osteoporosis who were followed in routine clinical practice were considered for inclusion. Clinical records of patients followed between January 2021 and December 2024 were retrospectively reviewed. Case identification was based on institutional electronic medical records and routinely maintained follow-up datasets, which were cross-checked to ensure data consistency and completeness. Patients were included if they had a confirmed diagnosis of osteoporosis, available baseline and follow-up clinical data, and sufficient follow-up to allow assessment of vertebral fracture outcomes and pain progression. Pain was evaluated using the Visual Analog Scale (VAS), as recorded during routine clinical visits. Patients with secondary causes of osteoporosis, incomplete clinical records, or insufficient follow-up were excluded. In addition, individuals receiving alternative bone-active therapies during the study period were not included in order to reduce treatment-related heterogeneity. Patients were categorized according to treatment exposure into those receiving ibandronate and those managed without ibandronate, reflecting routine clinical decision-making rather than protocol-driven allocation.

**Figure 1:**
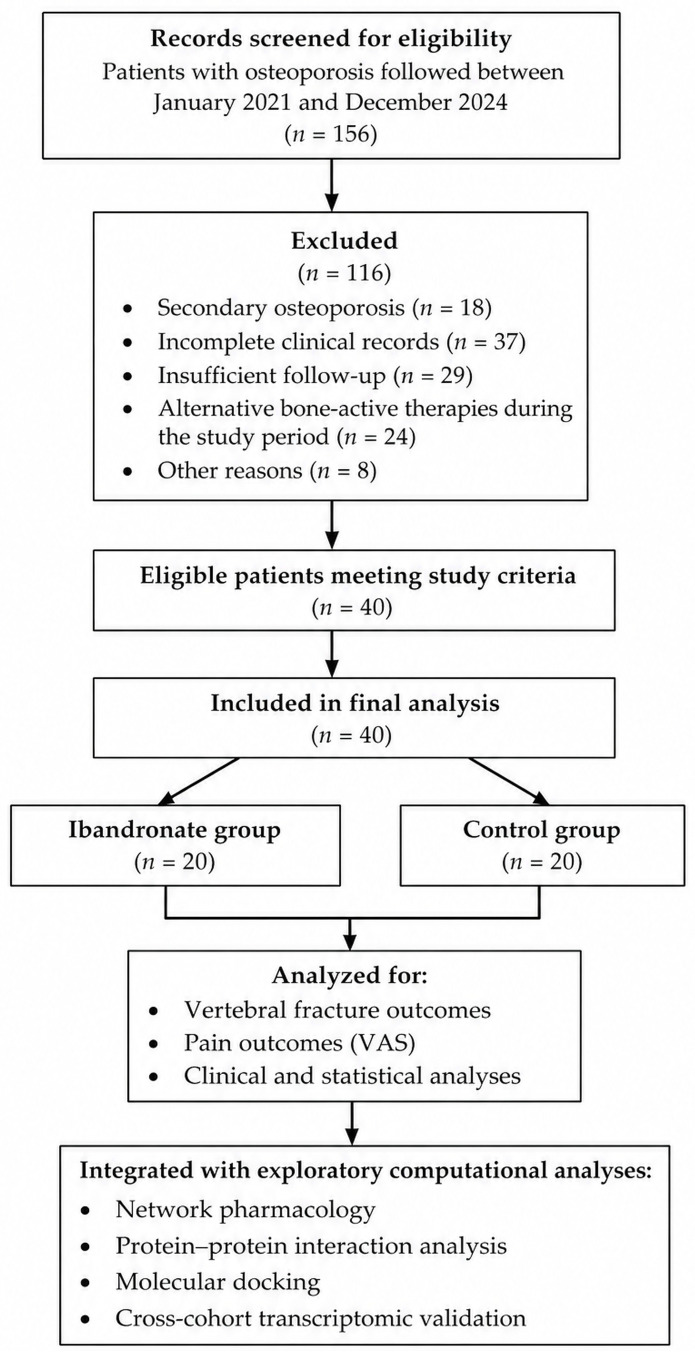
STROBE flow diagram of patient screening, eligibility assessment, and study inclusion.

A total of 40 patients met the study criteria, including 20 patients in the ibandronate group and 20 in the control group. Details of patient screening, eligibility assessment, and final inclusion are presented in the STROBE flow diagram (Figure 1).

### 2.3. Treatment Exposure

Treatment exposure was defined according to documented use of oral ibandronate in routine clinical practice. All patients in the treatment group received a standard oral dose of 150 mg once monthly. Information regarding treatment initiation, duration, and adherence was obtained from prescription records and clinical follow-up documentation. The duration of ibandronate use varied among patients, reflecting real-world prescribing patterns rather than a fixed treatment protocol. Patients were classified as exposed if they had documented initiation of ibandronate therapy with continued use during the observation period. The timing of treatment initiation and total treatment duration (months) were recorded for all patients. The control group consisted of patients managed without ibandronate during the same observation period. These individuals received routine clinical management, including calcium and vitamin D supplementation when clinically indicated. Given the non-randomized design and variability in exposure duration, analyses were performed with consideration of potential confounding related to treatment exposure and observation time. Total follow-up duration was therefore incorporated into adjusted models to account for differences in observation periods between groups.

### 2.4. Outcomes and Definitions

The primary outcome of the study was the occurrence of new vertebral fractures during the follow-up period. Vertebral fractures were identified based on radiological evaluations performed as part of routine clinical care. A fracture was defined as a newly detected vertebral compression deformity not present on baseline imaging, as documented in radiology reports and interpreted according to established radiological definitions of vertebral compression fractures.

The secondary outcome was change in pain severity over time, assessed using Visual Analog Scale (VAS) scores recorded during routine outpatient follow-up. Change in pain (ΔVAS) was calculated as the difference between baseline and final available VAS measurements, with positive values corresponding to improvement in pain severity. Baseline VAS was defined as the first recorded pain score at study entry, whereas the follow-up value corresponded to the most recent available assessment during the observation period. Given the retrospective study design, follow-up intervals were not standardized and reflected routine clinical practice.

Radiological outcomes were additionally evaluated descriptively as progression or improvement based on follow-up imaging reports; however, these were not used as primary endpoints. Outcome data were obtained from institutional electronic medical records and cross-checked with follow-up documentation to ensure internal consistency.

### 2.5. Statistical Analysis

All statistical analyses were performed according to a prespecified analytical framework using IBM SPSS Statistics (Version 30; IBM Corp., Armonk, NY, USA). Continuous variables were evaluated for distributional characteristics using visual inspection and appropriate normality assessment methods. Depending on distribution, data are presented as mean ± standard deviation (SD) or median with interquartile range (IQR). Categorical variables are summarized as counts and percentages. Baseline characteristics between the ibandronate and control groups were compared using the independent samples *t*-test or Mann–Whitney U test for continuous variables, and the χ^2^ test or Fisher’s exact test for categorical variables, as appropriate.

The primary outcome, new vertebral fracture occurrence, was analyzed using logistic regression models. Crude associations were initially estimated, followed by multivariable adjustment for clinically relevant covariates, including age, lumbar spine bone mineral density (BMD) T-score, and total follow-up duration. These covariates were selected a priori based on their established clinical relevance to osteoporotic fracture risk and outcome assessment. Given the limited sample size and number of outcome events, model complexity was intentionally restricted to minimize the risk of overfitting. Additional sensitivity analyses further incorporated calcium/vitamin D use. Effect estimates are reported as odds ratios (ORs) with 95% confidence intervals (CIs). The secondary outcome, change in pain severity (ΔVAS), was evaluated using both unadjusted comparisons and multivariable linear regression models. The primary adjusted analysis included baseline VAS and age as covariates. Additional sensitivity analyses further incorporated total follow-up duration and calcium/vitamin D use. Results are presented as regression coefficients (β) with corresponding 95% CIs.

Within-group changes in VAS scores were additionally evaluated using paired *t*-tests or non-parametric equivalents, as appropriate according to data distribution. Given the non-randomized study design and the potential for confounding by indication, exploratory propensity score matching (PSM) analyses were performed as sensitivity analyses where feasible. In addition, overlap-weighted regression models based on propensity scores estimated from baseline demographic and clinical variables were constructed to further examine the consistency of the findings. Because of the limited sample size, reduced matching efficiency, and residual covariate imbalance, propensity-score-based analyses were interpreted cautiously and considered exploratory. Patients with incomplete clinical records were excluded during eligibility assessment; therefore, all analyses were conducted using complete-case data and no imputation procedures were applied.

All statistical tests were two-sided, and a *p*-value < 0.05 was considered statistically significant.

### 2.6. In Silico Analyses

To provide a mechanistic context for the clinical findings, an in silico workflow was constructed to examine the potential relationship between FDPS-centered osteoclast inhibition and the inflammatory pain axis. The molecular interactions of ibandronic acid evaluated in the present study were assessed on target proteins including FDPS (PDB ID: 1YV5) [9], CTSK (PDB ID: 1ATK) [10], MMP9 (PDB ID: 5I12) [11], COX-2 (PDB ID: 3LN1) [12], COX-1 (PDB ID: 3KK6) [13], TNF-α (PDB ID: 2AZ5) [14], IL-1β (PDB ID: 5R85) [15], MAPK14 (PDB ID: 1A9U) [16], VDR (PDB ID: 1DB1) [17], MAPK1 (PDB ID: 2OJJ) [18], NF-κB (PDB ID: 1LE9) [19], ACP5 (PDB ID: 2BQ8) [20], PTH1R (PDB ID: 6FJ3) [21], and SCN9A (PDB ID: 7XMF) [22]. Targets were included if they were considered biologically relevant to the hypothesized link between osteoclast activity and pain-related inflammatory signaling. Targets lacking sufficient biological relevance to this predefined framework were not included in downstream analyses. The systems-level analytical workflow was constructed sequentially using transcriptomic and network-based approaches. Differentially expressed genes identified across the GEO cohorts were first evaluated together with literature-supported osteoporosis-, inflammatory-, and pain-related targets. The resulting expanded target panel was subsequently used for STRING-based PPI construction, Cytoscape topology analysis, and disease-enrichment analyses. Molecular docking analyses were then performed as a complementary exploratory structural approach to assess the potential ligand–target interaction profiles of selected biologically relevant proteins.

#### 2.6.1. Molecular Docking

During the protein training stage, crystallographic water molecules associated with the target protein structures were removed, polar hydrogen atoms were added, and the required atomic charges were assigned to ensure compatibility with the docking procedure. Following ligand and receptor preparation, molecular docking analyses were performed using the Lamarckian Genetic Algorithm (LGA) implemented in AutoDock 4.2.6 [23]. For each ligand–protein pair, forty independent docking runs were conducted to evaluate potential binding conformations within a broad conformational sampling framework. The resulting binding poses were analyzed according to binding energies, hydrogen bonding patterns, structural compatibility with the active site, and root mean square deviation (RMSD) values. Conformations with RMSD values ≤ 2 Å were considered stable and consistent binding modes.

Interaction analyses were performed using BIOVIA Discovery Studio Visualizer (2025 Client). Conventional hydrogen bonds, attractive charge interactions (anionic/cationic electrostatic interactions), alkyl/pi-alkyl interactions, and pi-sigma interactions within ligand–protein complexes were evaluated in detail. Each binding pose was assessed not only in terms of binding energy but also according to its structural compatibility with the active site and the presence of these fundamental non-covalent interaction patterns. Two-dimensional (2D) interaction diagrams and 3D binding conformations were generated to evaluate ligand orientation within the binding pocket, contacts with interacting amino acid residues, and structural features potentially contributing to binding compatibility.

#### 2.6.2. PPI Network Analysis

PPI analysis was performed using the STRING database (version 12.0; available online: https://string-db.org/; accessed on 10 May 2026) [24]. A high-confidence interaction score threshold (≥0.700) was applied to ensure stringent interaction filtering. Active interaction sources were limited to experimentally validated interactions and curated databases. No additional interactors were included, and the network was restricted to the predefined target set. The resulting network was evaluated in terms of overall connectivity and interaction enrichment. Network parameters, including the number of nodes and edges, average node degree, and PPI enrichment *p*-value, were recorded.

#### 2.6.3. Network Visualization and Topological Analysis

The PPI network obtained from the STRING database was imported into Cytoscape (version 3.10.4; available online: https://cytoscape.org/; accessed on 10 May 2026) [25] for visualization and topological analysis. Network topology parameters were calculated using the NetworkAnalyzer tool implemented in Cytoscape [26]. Key centrality measures, including node degree and betweenness centrality, were evaluated to characterize the structural properties of the network and to identify highly connected nodes (hub genes). Hub gene analysis was additionally performed using the cytoHubba plugin (version 0.1; available online: http://apps.cytoscape.org/apps/cytohubba; accessed on 10 May 2026) [27] based on the maximal clique centrality (MCC) algorithm. MCC scores were extracted directly from cytoHubba output and reported as raw values without normalization or transformation. For visualization, a force-directed layout was applied to improve network interpretability. Nodes were visually mapped according to their degree values, and hub genes were highlighted to facilitate interpretation of the network structure.

#### 2.6.4. Disease Enrichment Analysis

Disease enrichment analysis was performed using the Enrichr platform developed by the Ma’ayan Laboratory (available online: https://maayanlab.cloud/Enrichr/; accessed on 10 May 2026) [27]. The network-pharmacology target panel was used as input, and enrichment was assessed against disease-associated gene sets derived from the DisGeNET database [28]. Disease–gene associations in DisGeNET are curated from multiple sources, including expert-curated repositories, genome-wide association studies, and text-mined literature, providing a comprehensive representation of disease-related genomic information. In addition, disease-gene association data were interpreted in the context of integrative resources such as the DISEASES database [29]. Enriched disease terms were ranked according to adjusted *p*-values, and biologically relevant categories related to bone metabolism, inflammatory processes, and pain-related pathways were prioritized for interpretation.

### 2.7. Exploratory Transcriptomic Comparison Across GEO Cohorts

Exploratory transcriptomic comparison was performed using three publicly available microarray cohorts obtained from the Gene Expression Omnibus (GEO): a primary discovery dataset (GSE230665) [30], an independent transcriptomic cohort (GSE35958) [31], and an exploratory peripheral blood B-cell dataset (GSE7429) [32] included as a supplementary orthogonal comparator. The three cohorts were intentionally selected to differ in tissue context and platform characteristics so that any observed cross-dataset concordance would be less likely to reflect platform-specific artefacts alone.

#### 2.7.1. Datasets and Platforms

GSE230665 [30] was used as the primary discovery cohort. In the uploaded GEO2R/limma workflow, this dataset was analysed on platform GPL10332 and included 15 samples, comprising 3 controls and 12 postmenopausal osteoporosis (PMO) samples. GSE35958 [31] was used as an independent transcriptomic cohort. In the uploaded GEO2R/limma workflow, this dataset was analysed on platform GPL570 and included 9 samples, comprising 4 controls and 5 PMO samples. GSE7429 [32] was included only as an exploratory peripheral blood B-cell comparator. In the uploaded GEO2R/limma workflow, this dataset was analysed on platform GPL96 and included 20 samples, comprising 10 high-BMD controls and 10 low-BMD samples. Because this cohort differs substantially from the bone- and hMSC-based cohorts in tissue and cellular context, it was not interpreted as confirmatory validation.

#### 2.7.2. Differential-Expression Analysis and DEG Threshold

For each cohort, differential-expression analysis was performed independently using the GEO2R platform, which applies the limma package for empirical Bayes–moderated linear modelling. Group assignments were defined according to the GEO2R workflows for each dataset. For GSE230665 and GSE35958, the comparison was defined as Control−PMO, whereas for GSE7429 the comparison was defined as high-BMD−low-BMD. Accordingly, positive log2 fold-change values indicate relatively higher expression in controls (or high-BMD samples), whereas negative log2 fold-change values indicate relatively higher expression in PMO (or low-BMD samples). To maintain methodological consistency across cohorts, a unified differential-expression threshold of |log2 fold-change| ≥ 1.0 and Benjamini–Hochberg adjusted *p*-value < 0.05 was applied to all datasets.

#### 2.7.3. Cross-Cohort Concordance Analysis

Cross-cohort concordance analysis was performed between GSE230665 and GSE35958, whereas GSE7429 was evaluated separately as an exploratory peripheral blood comparator and is presented in Supplementary Section S4. The comparison universe was defined according to the shared HUGO Gene Nomenclature Committee (HGNC) gene symbols identified across both cohorts (*n* = 17,180). Within this shared gene set, overlapping differentially expressed genes (DEGs) were further classified according to concordant directionality (same sign of log2 fold-change across cohorts) or discordant directionality (opposite signs across cohorts). To assess whether the observed DEG overlap exceeded random expectation, a one-sided hypergeometric test and observed/expected overlap ratio were calculated using the shared gene universe.

#### 2.7.4. Hub-Gene Cross-Cohort Assessment

The thirteen hub genes identified in the network pharmacology analyses (MAPK1, CTNNB1, MMP9, VDR, TNF, MAPK14, RUNX2, IL6, IL1B, NFKB1, PTGS2, TNFSF11, and TRPV1) were evaluated across the differential-expression results of GSE230665 and GSE35958 using HGNC gene symbols. For each hub gene, log2 fold-change, adjusted *p*-value, and DEG status under the unified threshold were recorded separately for each cohort. Hub genes were subsequently categorized according to their cross-cohort expression patterns as concordant (significant in both cohorts with the same direction of change), discordant (significant in both cohorts with opposite directions), discovery-only (significant only in GSE230665), independent-cohort-only (significant only in GSE35958), not detected (not significant in either cohort), or absent on platform. All directional interpretations were based on the predefined Control−PMO comparison framework.

#### 2.7.5. Software

Differential-expression and concordance analyses were performed using GEO2R/limma-based workflows together with standard statistical procedures in R (version 4.6.0; R Foundation for Statistical Computing, Vienna, Austria). Visualizations were generated in Python using matplotlib.

## 3. Results

### 3.1. Study Population and Baseline Characteristics

A total of 40 patients were included in the analysis, comprising 20 patients in the ibandronate group and 20 patients in the control group.

Baseline characteristics of the study population are summarized in Table 1, with additional baseline, laboratory, and clinical variables provided in Supplementary Table S1.

There were no significant differences between groups in terms of age (63.65 ± 10.14 vs. 67.20 ± 8.64 years, *p* = 0.241), body mass index (27.61 ± 4.32 vs. 27.15 ± 4.67 kg/m^2^, *p* = 0.748), or years since menopause (16.00 ± 7.00 vs. 16.60 ± 9.44, *p* = 0.821).

Similarly, bone mineral density parameters, including lumbar spine T-score (median −2.41 vs. −2.23, *p* = 0.766), did not differ significantly between groups. The prevalence of prior fractures (60% vs. 65%, *p* = 1.000), smoking status (30% vs. 20%, *p* = 0.716), and comorbidity burden (70% in both groups, *p* = 1.000) did not differ significantly between the groups.

However, the control group had a significantly longer follow-up duration (median 46 vs. 33 months, *p* = 0.021), and calcium/vitamin D supplementation was more frequent in the control group (70% vs. 30%, *p* = 0.026). Baseline pain scores were numerically higher in the ibandronate group, although this difference did not reach statistical significance (median VAS 6 vs. 5, *p* = 0.071).

**Figure 2:**
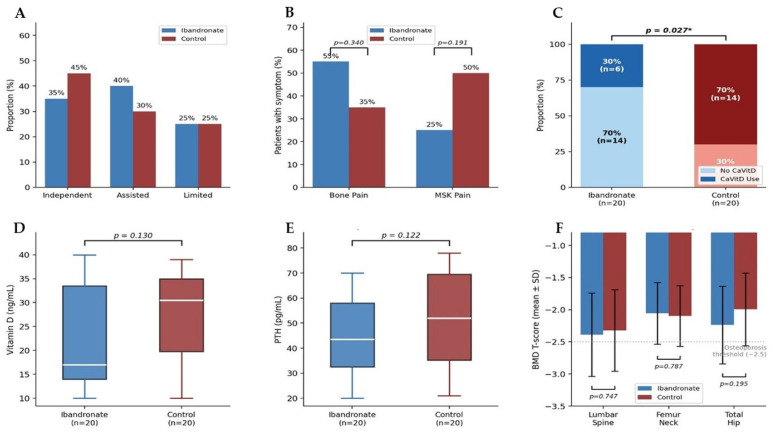
Clinical and laboratory baseline characteristics by treatment group. (**A**) Mobility status distribution. (**B**) Proportion of patients reporting bone pain and musculoskeletal pain. (**C**) Calcium and vitamin D supplementation use. (**D**) Serum vitamin D levels (ng/mL). (**E**) Parathyroid hormone (PTH) levels (pg/mL). (**F**) Bone mineral density T-scores at the lumbar spine, femur neck, and total hip. Data are presented as proportions (%) or mean ± SD. *p*-values are derived from χ^2^ test or independent samples *t*-test, as appropriate. Asterisk (*) in panel (**C**) denotes statistical significance (*p* <0.05). In panel (**C**), the darker-colored segments represent patients receiving calcium/vitamin D supplementation, whereas the lighter-colored segments represent patients without supplementation.

The distribution of selected clinical and laboratory baseline parameters across groups is further illustrated in Figure 2, including mobility status, pain symptoms, calcium/vitamin D supplementation, serum vitamin D and parathyroid hormone levels, and bone mineral density T-scores. Notably, calcium/vitamin D supplementation was significantly more frequent in the control group (70% vs. 30%, *p* = 0.026), while other parameters shown in Figure 2 did not differ significantly between groups.

### 3.2. Primary Outcome: New Vertebral Fractures

**Figure 3:**
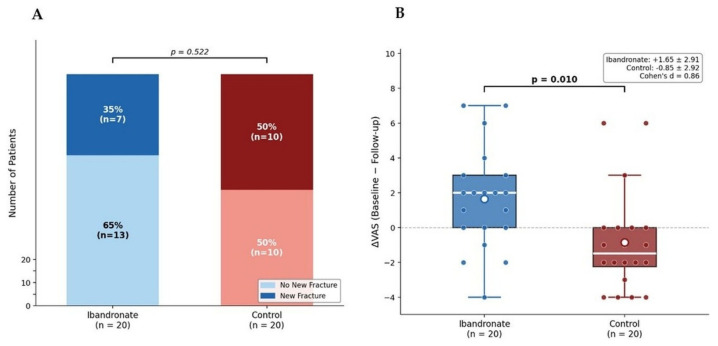
Primary and secondary clinical outcomes by treatment group. (**A**) Incidence of new vertebral fractures during follow-up. A lower proportion of new fractures was observed in the ibandronate group; however, the difference was not statistically significant (*p* = 0.522). (**B**) Change in pain scores (ΔVAS) according to treatment group. The ibandronate group showed a greater unadjusted reduction in pain compared with controls, with a statistically significant unadjusted between-group difference (*p* = 0.010). In panel (**A**), darker-colored segments represent patients with new vertebral fractures, whereas lighter-colored segments represent patients without new vertebral fractures.

During follow-up, new vertebral fractures occurred in 7 patients (35%) in the ibandronate group and 10 patients (50%) in the control group. This difference was not statistically significant in unadjusted analysis (*p* = 0.522) (Table 2, Figure 3A).

In crude logistic regression analysis, ibandronate use was associated with a reduction in the odds of new vertebral fracture; however, this association was not statistically significant (odds ratio [OR] 0.54, 95% confidence interval [CI] 0.15–1.92, *p* = 0.339). After adjustment for age, lumbar spine BMD T-score, and total follow-up duration, the point estimate remained consistent; however, the association remained statistically non-significant (adjusted OR 0.37, 95% CI 0.08–1.67, *p* = 0.197; Supplementary Table S2).

### 3.3. Secondary Outcome: Change in Pain (ΔVAS)

Change in pain was assessed as the difference between baseline and follow-up VAS scores (ΔVAS). The association was attenuated and was no longer statistically significant after adjustment. Patients in the ibandronate group showed a greater unadjusted reduction in pain compared with the control group (mean ΔVAS 1.65 ± 2.91 vs. −0.85 ± 2.92). The unadjusted mean between-group difference was 2.50 points (95% CI 0.63–4.37; *p* = 0.010), corresponding to a large effect size (Cohen’s d = 0.86) (Figure 3B).

However, in adjusted linear regression analysis controlling for baseline VAS and age, the association between ibandronate use and ΔVAS was attenuated and no longer statistically significant (adjusted β = 1.12, 95% CI −0.27 to 2.51, *p* = 0.110). Baseline VAS was a strong independent predictor of pain reduction (*p* < 0.001) (Table 3).

Within-group changes in pain scores are presented in Supplementary Table S3. Propensity score matching was performed as a sensitivity analysis; results are presented in Supplementary Table S4.

### 3.4. Radiological Outcomes

Radiological progression was descriptively observed in 60% of patients in the ibandronate group and 55% of patients in the control group. Separately, radiological improvement was documented in 40% and 60% of patients, respectively (*p* = 0.343 for between-group comparison of improvement rates). Because radiological progression and partial radiological improvement were not defined as mutually exclusive categories in routine clinical records, some patients were classified as showing both findings during follow-up imaging assessments. Given the lack of standardized radiological scoring and the exploratory nature of these observations, these findings should be interpreted cautiously (Figure S1).

Propensity score matching was conducted as a sensitivity analysis; however, due to the limited sample size, reduced matching efficiency, and residual covariate imbalance, the findings were considered exploratory and are presented in the Supplementary Materials.

Overlap weighting improved covariate balance, with all standardized mean differences reduced to <0.05 (Supplementary Table S7). Sensitivity analyses incorporating additional adjustment for calcium/vitamin D use and follow-up duration, as well as overlap-weighted models, showed generally similar effect estimates without reaching statistical significance (Supplementary Tables S5–S8).

### 3.5. In Silico Results

#### 3.5.1. Molecular Docking Results

Molecular docking analyses showed variable binding affinities of ibandronic acid across the selected target proteins (Supplementary Table S9). Among the evaluated complexes, lower binding-energy values were observed for MMP9, IL-1β, VDR, COX-2, and COX-1, consistent with potentially relevant ligand–target interactions. Representative docking poses were selected within the predefined RMSD clustering threshold.

**Figure 4:**
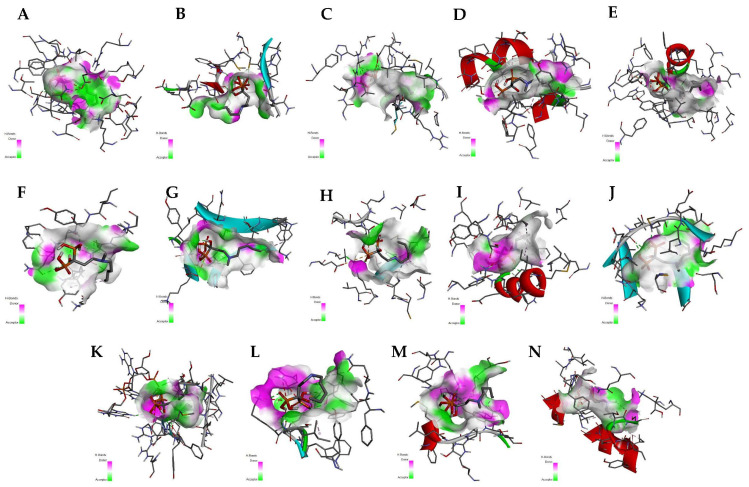
Binding conformations of ibandronic acid within the binding regions of the selected target proteins. Ligands are displayed in stick representation, whereas proteins are shown using surface models. Surface color mapping illustrates putative hydrogen bond donor and acceptor regions within the binding pocket. (**A**) FDPS–ibandronic acid, (**B**) CTSK–ibandronic acid, (**C**) MMP9–ibandronic acid, (**D**) COX-2–ibandronic acid, (**E**) COX-1–ibandronic acid, (**F**) TNF-α–ibandronic acid, (**G**) IL-1β–ibandronic acid, (**H**) MAPK14–ibandronic acid, (**I**) VDR–ibandronic acid, (**J**) MAPK1–ibandronic acid, (**K**) NF-κB–ibandronic acid, (**L**) ACP5–ibandronic acid, (**M**) PTH1R–ibandronic acid, and (**N**) SCN9A–ibandronic acid.

**Figure 5:**
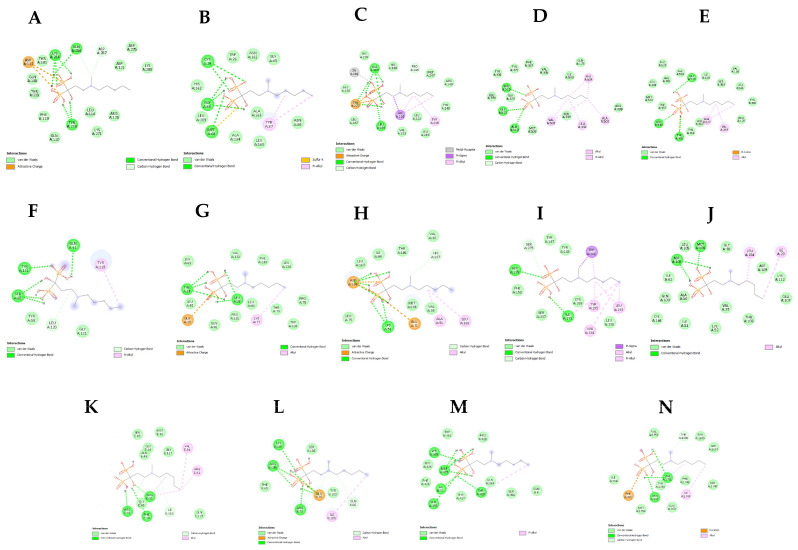
Interaction diagrams of the ibandronic acid–protein complexes. Dashed interaction lines and color-coded interaction patterns represent different classes of non-covalent interactions identified during docking analysis. (**A**) FDPS–ibandronic acid, (**B**) CTSK–ibandronic acid, (**C**) MMP9–ibandronic acid, (**D**) COX-2–ibandronic acid, (**E**) COX-1–ibandronic acid, (**F**) TNF-α–ibandronic acid, (**G**) IL-1β–ibandronic acid, (**H**) MAPK14–ibandronic acid, (**I**) VDR–ibandronic acid, (**J**) MAPK1–ibandronic acid, (**K**) NF-κB–ibandronic acid, (**L**) ACP5–ibandronic acid, (**M**) PTH1R–ibandronic acid, and (**N**) SCN9A–ibandronic acid.

Interaction analyses identified multiple conventional hydrogen bonds, attractive charge interactions, alkyl/pi-alkyl contacts, and pi-sigma interactions across the evaluated ligand–protein complexes (Figure 4 and Figure 5). The 2D chemical structure of ibandronic acid is provided in Supplementary Figure S2.

#### 3.5.2. PPI Network and Functional Enrichment Analysis

The PPI network constructed from the network-pharmacology target panel showed a higher level of connectivity than expected by chance. The network consisted of 24 nodes and 69 edges, with an average node degree of 5.75, whereas the expected number of edges was 9, yielding a PPI enrichment *p*-value < 1.0 × 10^−16^.

Network topology analysis identified several highly connected nodes, including TNFSF11 (degree = 12), IL6 (11), CTNNB1 (11), IL1B (10), TNF (10), and RUNX2 (10), consistent with relatively higher network connectivity. In contrast, FDPS, CYP27B1, and PTH1R exhibited no direct interactions under the applied high-confidence threshold, consistent with more limited connectivity under the selected network parameters.

GO Biological Process enrichment analysis identified significant enrichment of pathways related to bone formation and remodeling, including ossification (13 genes, FDR = 6.06 × 10^−14^) and bone remodeling (6 genes, FDR = 2.82 × 10^−8^). In parallel, enrichment was additionally observed in inflammatory processes, such as positive regulation of acute inflammatory response (FDR = 3.24 × 10^−9^) and regulation of inflammatory response (FDR = 1.11 × 10^−8^), as well as responses to external and biochemical stimuli, including response to lipopolysaccharide (FDR = 3.32 × 10^−10^).

Additional enrichment patterns involved osteoblast differentiation, skeletal system development, and bone resorption, consistent with enrichment of bone-remodeling-related pathways within the network. Molecular function analysis identified enrichment in cytokine activity and prostaglandin-endoperoxide synthase activity, corresponding to inflammatory mediators including IL1B, IL6, TNF, PTGS1, and PTGS2.

KEGG pathway analysis showed enrichment in osteoclast differentiation (10 genes, FDR = 6.63 × 10^−14^), together with inflammatory signaling pathways, including IL-17 signaling (FDR = 3.49 × 10^−11^) and TNF signaling (FDR = 1.05 × 10^−10^). Additional enrichment was observed in Toll-like receptor and C-type lectin receptor signaling pathways, as well as in disease-associated pathways including rheumatoid arthritis. These pathways shared overlapping gene components, including MAPK1, MAPK14, NFKB1, IL1B, IL6, and TNF. These enrichment results are summarized in Table 4.

Reactome pathway analysis additionally identified enrichment in cytokine signaling in the immune system (11 genes, FDR = 5.03 × 10^−7^) and interleukin-mediated signaling pathways, including IL-4, IL-10, and broader interleukin signaling pathways.

**Figure 6:**
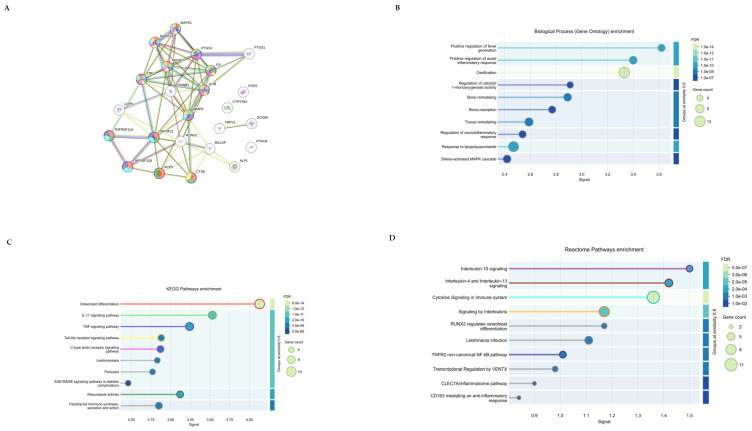
PPI network and functional enrichment analysis of the network-pharmacology target panel. (**A**) PPI network constructed using the STRING database, illustrating the interactions among the predefined targets. Nodes represent proteins and edges correspond to functional associations. (**B**) Gene Ontology (Biological Process) enrichment analysis showing the top enriched biological processes. (**C**) KEGG pathway enrichment analysis highlighting pathways related to osteoclast differentiation and inflammatory signaling. (**D**) Reactome pathway enrichment analysis showing enrichment in cytokine-mediated and interleukin-related signaling pathways. Bubble size represents gene count, whereas color intensity corresponds to the level of statistical significance based on false discovery rate (FDR).

Overall, the enrichment profile was centered around osteoclast differentiation, inflammatory signaling, and cytokine-mediated pathways. The PPI network and associated functional enrichment analyses are summarized in Figure 6A–D.

Additional Gene Ontology (Molecular Function) enrichment results are provided in Supplementary Figure S3 to complement the functional analysis presented above.

#### 3.5.3. Cytoscape Network Topology Analysis

Topological analysis of the Cytoscape-derived interaction network identified several highly connected nodes within the central cluster. Based on degree centrality, TNFSF11, IL6, CTNNB1, IL1B, TNF, and RUNX2 were among the most connected genes, consistent with relatively higher topological connectivity within the network. To further refine hub-gene prioritization, the cytoHubba plugin was applied using the MCC algorithm. NetworkAnalyzer-derived topological parameters of the MCC-selected hub subnetwork are summarized in Table 5.

MCC-based ranking identified IL6, TNF, and NFKB1 as the highest-ranked hub proteins, followed by CTNNB1, PTGS2, IL1B, MAPK14, MMP9, MAPK1, and TNFSF11 (Table 6).

Several nodes, including FDPS, CYP27B1, and PTH1R, exhibited minimal or no connectivity under the applied confidence threshold, consistent with relatively peripheral positions within the network. The Cytoscape network and top 10 hub-gene subnetwork are shown in Supplementary Figure S4.

#### 3.5.4. Disease Enrichment Analysis

Disease enrichment analysis using the DisGeNET database identified enrichment of bone-related and inflammatory disease categories. The most significantly enriched terms included osteoporosis, osteopenia, bone diseases, and PMO, consistent with bone remodeling and osteoclast-related processes. In addition, inflammatory musculoskeletal conditions such as rheumatoid arthritis, arthritis, and degenerative polyarthritis were also enriched, compatible with partial overlap between bone metabolism and inflammatory signaling pathways.

Pain-related phenotypes, including hyperalgesia and related nociceptive conditions, were also represented, with contributing genes such as IL6, TNF, IL1B, PTGS2, and TRPV1 appearing across multiple enriched terms. Overall, the enriched disease terms involved bone remodeling, inflammatory signaling, and pain-related pathways. The top enriched disease terms identified from the network-pharmacology target panel are summarized in Table 7.

The disease enrichment results are additionally visualized in Supplementary Figure S5.

### 3.6. Independent Transcriptomic Analyses Across GEO Cohorts

#### 3.6.1. Discovery–Validation Concordance

Under the unified DEG threshold (|log2 fold-change| ≥ 1.0 and adj.P.Val < 0.05) and the identical Control − PMO contrast applied across both cohorts, the discovery dataset (GSE230665) and the external validation dataset (GSE35958) yielded 1388 shared differentially expressed genes from a shared universe of 17,180 HGNC symbols present on both platforms after probe-to-symbol collapse. The observed overlap exceeded that expected under independent DEG calls (observed/expected = 1.20; one-sided hypergeometric *p* = 2.06 × 10^−26^), supporting cross-cohort concordance at the gene-set level.

**Figure 7:**
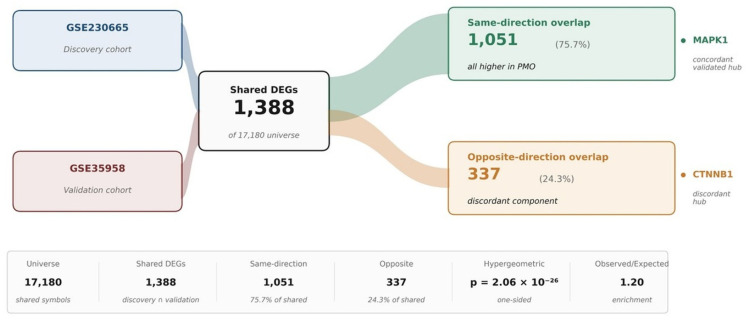
Discovery–validation concordance of differentially expressed genes between the discovery cohort (GSE230665) and the external validation cohort (GSE35958) using a unified Control−PMO contrast definition across both cohorts. Differential expression analysis was performed under the same DEG significance criteria (|log2 fold-change| ≥ 1.0 and adjusted *p* value < 0.05), enabling direct cross-cohort comparison and validation concordance assessment.

A detailed visualization of differential expression distributions and cross-cohort DEG overlap is provided in Supplementary Figure S6. Of the 1388 shared DEGs, 1051 (75.7%) showed the same direction of effect across both cohorts, whereas 337 (24.3%) showed opposite directions. Under the Control−PMO sign convention, the same-direction subset predominantly reflected genes with relatively higher expression in PMO across both datasets. The opposite-direction subset is reported transparently and was not consolidated into a single directional category (Figure 7; Supplementary Tables S10–S12).

Under the same analytical framework, the peripheral blood B-cell cohort (GSE7429) was included as an exploratory comparator, and the full gene-level differential expression results are provided in Supplementary Table S13.

#### 3.6.2. Hub-Gene Cross-Cohort Status

**Figure 8:**
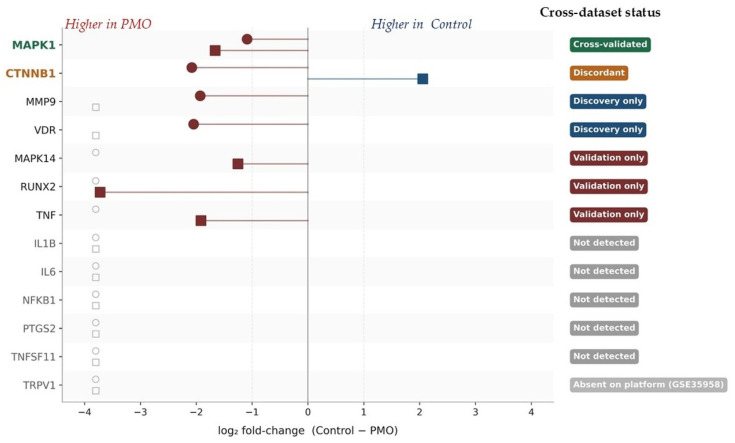
Hub-gene cross-cohort status across the discovery cohort (GSE230665) and the external validation cohort (GSE35958). Per-cohort log2 fold-change values and DEG significance status are shown for the thirteen network-pharmacology hub genes (MAPK1, CTNNB1, MMP9, VDR, TNF, MAPK14, RUNX2, IL6, IL1B, NFKB1, PTGS2, TNFSF11, and TRPV1), together with the pre-specified cross-cohort classification framework used to assess concordance and validation consistency between cohorts. Filled markers denote statistically significant differential expression under the unified DEG threshold (|log2FC| ≥ 1.0 and adjusted *p* value < 0.05), whereas open markers indicate non-significant or undetected genes. Red color coding indicates higher expression in PMO samples, blue indicates higher expression in Control samples, and grey denotes genes not detected or absent in the corresponding dataset. Circular markers represent the discovery cohort (GSE230665), whereas square markers represent the external validation cohort (GSE35958).

The thirteen hub genes nominated by the upstream network-pharmacology analysis were queried against the gene-level differential-expression statistics of GSE230665 and GSE35958 under the same unified threshold. MAPK1 reached the DEG threshold in both cohorts with the same direction of effect and was therefore classified as a same-direction cross-validated hub gene. CTNNB1 also reached the DEG threshold in both cohorts but showed opposite directions of effect across the two datasets and was therefore categorized as discordant. Several additional hub genes reached the DEG threshold in only one cohort, including MMP9 and VDR in GSE230665 and TNF, MAPK14, and RUNX2 in GSE35958. The remaining hub genes (IL6, IL1B, NFKB1, PTGS2, and TNFSF11) did not meet the predefined DEG threshold in either cohort, whereas TRPV1 was not represented on the GSE35958 platform after probe-to-symbol collapse and was therefore annotated as absent on platform (Figure 8).

The per-cohort log2 fold-change distributions of the thirteen hub genes across all datasets are visualized in Supplementary Figure S7.

Overall, these findings suggest partial cross-cohort reproducibility of the network-pharmacology hub-gene panel at the differential-expression level under identical statistical criteria applied across two independent bone-context PMO transcriptomic cohorts.

Taken together, the discovery and validation analyses provide cautious, hypothesis-generating support for the network-pharmacology framework. A highly significant and predominantly same-direction DEG overlap was observed between GSE230665 and GSE35958; however, only one prioritized hub gene (MAPK1) met the predefined DEG threshold in both cohorts with the same direction of effect. The remaining hub genes showed single-cohort significance, discordance (CTNNB1), or no detectable differential expression under the applied criteria. These observations are compatible with the possibility that inducible inflammatory cytokine-related hub genes (e.g., IL6, IL1B, TNF, PTGS2, TNFSF11, NFKB1) may not be consistently represented as DEGs in resting bulk-tissue transcriptomic datasets without stimulus-specific or cell-type-resolved context.

Detailed per-gene cross-cohort expression profiles and classification of the thirteen hub genes are provided in Supplementary Table S14.

## 4. Discussion

In this retrospective analysis, ibandronate use was associated with a numerically lower incidence of new vertebral fractures; however, this difference did not reach statistical significance. In contrast, a greater reduction in pain scores was observed in the ibandronate group in unadjusted analyses, although this association was attenuated after adjustment for baseline pain levels.

The lack of statistical significance in fracture outcomes may be explained by the limited sample size and the relatively low number of events, which reduce statistical power. The direction of the association, however, remained consistent across crude and adjusted models, suggesting a potential effect that could not be confirmed within the current sample. Recent therapeutic updates and large-scale analyses have consistently supported the anti-fracture efficacy of bisphosphonates, although variability in treatment response and the influence of patient-level factors remain important considerations in real-world settings [2,4]. In this context, the absence of a statistically significant reduction in fracture incidence in the present study may reflect limited statistical power and heterogeneity inherent to real-world cohorts rather than a lack of biological effect. Importantly, the consistent direction of association observed across crude and adjusted models remains broadly aligned with the existing literature suggesting a protective effect of antiresorptive therapy. Similar challenges in interpreting fracture outcomes have been reported in recent real-world osteoporosis cohorts, where treatment allocation was based on clinical judgment rather than randomization and fracture events remained relatively infrequent despite active antiresorptive therapy. In such settings, residual confounding and limited event numbers may contribute substantially to uncertainty in treatment-effect estimation [33]. Consistent with these observations, a recent retrospective study of patients with osteoporotic vertebral fractures identified bone mineral density and bone turnover markers as independent predictors of recurrent fracture risk, further highlighting the multifactorial determinants of fracture outcomes in real-world osteoporosis populations [34].

Emerging evidence suggests that pain trajectories in osteoporosis are not solely determined by structural changes, but may also reflect baseline symptom burden and inflammatory activity, contributing to variability in symptomatic response across patients [3,5]. Recent multi-omics and systems-biology frameworks have further suggested that osteoporosis progression and symptom manifestation may be influenced by complex interactions among chronic low-grade inflammation, oxidative stress, immune signaling, and bone-remodeling pathways, extending beyond structural bone deterioration alone [35]. In the present study, the attenuation of the association between ibandronate use and pain reduction after adjustment for baseline VAS may be interpreted as consistent with the possibility that initial symptom severity plays a dominant role in shaping observed clinical trajectories. The initially observed difference in pain reduction between groups was attenuated after adjustment, with baseline VAS emerging as a strong independent predictor of ΔVAS. Taken together, these findings suggest that the observed improvement in pain may be influenced, at least in part, by baseline symptom severity rather than treatment effect alone. The higher frequency of calcium/vitamin D supplementation in the control group further supports the likelihood of residual treatment heterogeneity despite multivariable and sensitivity-adjusted analyses. A similar degree of therapeutic heterogeneity has been reported in recent real-world postmenopausal osteoporosis cohorts, where substantial variation in pharmacological treatment patterns and supportive therapies was observed, reflecting the complexity of routine clinical decision-making and the challenges of interpreting treatment effects outside randomized settings [36]. This underscores the importance of accounting for baseline imbalance when interpreting treatment-related changes in patient-reported outcomes. Age was not significantly associated with changes in pain scores, and the effect size was minimal, suggesting that age did not meaningfully influence treatment response in this cohort. These findings are consistent with a recent prospective real-life study demonstrating significant improvement in back pain during osteoporosis treatment, while also showing that symptomatic trajectories remained heterogeneous across patients despite active pharmacological therapy, emphasizing the contribution of patient-specific factors beyond treatment allocation alone [37].

From a clinical perspective, these findings suggest that symptomatic improvement and structural outcomes may not necessarily evolve in parallel in patients with osteoporotic vertebral fractures. Accordingly, assessment of treatment response may benefit from consideration of both patient-reported outcomes and radiological findings rather than reliance on a single outcome domain alone. Although the present study does not permit definitive conclusions regarding treatment effectiveness, the observed divergence between pain-related and structural outcomes highlights the complexity of evaluating treatment response in routine osteoporosis care.

Notably, radiological outcomes did not parallel changes in pain scores, suggesting a discordance between structural and symptomatic responses. This observation may be considered within a broader clinical framework in which radiological improvement does not necessarily translate into symptomatic benefit, and is consistent with the view that pain in osteoporotic conditions reflects a multifactorial process extending beyond structural changes alone. In this regard, the dissociation between radiological and symptomatic trajectories may represent a clinically relevant feature rather than an unexpected inconsistency.

The exploratory transcriptomic analyses across independent GEO cohorts further supported the possibility that inflammatory and bone-remodeling pathways may converge within the broader biological context of PMO. Cross-cohort concordance between the primary discovery cohort (GSE230665) and the independent transcriptomic cohort (GSE35958) identified partially overlapping differentially expressed genes despite differences in platform characteristics and tissue context. In contrast, the peripheral blood B-cell dataset (GSE7429) showed limited concordance under the same stringent DEG thresholds, a finding we interpret cautiously as likely reflecting tissue- and cell-type heterogeneity rather than direct contradiction of the bone-associated transcriptomic signal.

Importantly, the observed cross-cohort concordance should be interpreted within the limitations of heterogeneous tissue sources, independent preprocessing pipelines, and relatively small cohort sizes, and therefore does not establish causal or treatment-specific molecular effects.

From a pharmacological perspective, the present findings may be interpreted within a broader biological framework involving osteoclast-related bone remodeling, inflammatory signaling, and nociceptive processes. Although ibandronate primarily acts through inhibition of osteoclast-mediated bone resorption, pain trajectories in osteoporotic vertebral fractures are unlikely to be determined exclusively by structural skeletal changes alone. Experimental and translational studies have suggested that cytokine-associated inflammatory pathways may contribute to both bone turnover and pain-related symptom generation, consistent with the biological plausibility of partially dissociated radiological and symptomatic trajectories. Within this context, the exploratory network-pharmacology and transcriptomic observations presented here should be interpreted as systems-level supportive findings rather than direct mechanistic evidence of ibandronate-specific molecular activity.

Consistent with this framework, the network-based analyses in the present study showed enrichment patterns involving cytokine signaling, interleukin cascades, and osteoclast differentiation. PPI and Cytoscape topology analyses identified several highly connected nodes, including IL6, TNF, NFKB1, and IL1B, which are well-recognized mediators of inflammatory signaling. Within this network structure, FDPS was located in a relatively peripheral position within the network under the applied thresholds, consistent with limited direct network connectivity within the constructed interaction map.

This observation may be interpreted cautiously as being consistent with the possibility that its influence on pain-related outcomes is indirect and potentially mediated through downstream pathways rather than through central network connectivity.

In parallel with the network-based findings, molecular docking analyses demonstrated heterogeneous binding profiles of ibandronic acid across multiple targets implicated in osteoclast regulation, inflammatory signaling, and nociceptive pathways. Relatively lower binding energy values were observed for several proteins involved in inflammatory and skeletal regulatory processes, including MMP9, IL-1β, VDR, COX-2, and COX-1, accompanied by structurally consistent docking poses within the predefined computational framework. Interaction analyses identified conventional hydrogen bonds, attractive charge interactions, alkyl/pi-alkyl contacts, and pi-sigma interactions across the evaluated ligand–protein complexes. Under the applied in silico conditions, these results suggest potential structural compatibility with multiple protein targets; however, given the reliance on static protein conformations and simplified computational assumptions, they should be interpreted as relative indicators of binding propensity rather than evidence of functional inhibition or pharmacological activity.

In a similar vein, certain targets, including FDPS and nociceptive ion channels, occupied relatively peripheral or weakly connected positions within the network, supporting the possibility of context-dependent or indirect roles under the applied analytical conditions. At a broader level, disease enrichment analysis identified enrichment patterns related to bone-metabolism-associated disorders, including osteoporosis and osteopenia, together with inflammatory and degenerative musculoskeletal conditions. Collectively, these enrichment patterns are compatible with partial overlap between inflammatory signaling and skeletal regulatory processes within the analyzed network, rather than evidence of a unified or directional mechanism. At the gene level, the co-occurrence of canonical inflammatory mediators (e.g., TNF, NFKB1, IL6, IL1B) with regulators of bone turnover (e.g., CTSK, ACP5, RUNX2, ALPL) may be considered consistent with the biological plausibility of an interface between immune activation and bone homeostasis. However, it is important to emphasize that these observations are derived from curated association datasets and network-based inference, and therefore do not establish causality or directionality. Accordingly, the present findings should be interpreted as exploratory and hypothesis-generating rather than confirmatory. While the observed enrichments are compatible with a network organized around inflammatory signaling and bone remodeling pathways, they do not permit inference regarding effect size, temporal relationships, or tissue-specific mechanisms.

This study has several limitations. First, the retrospective design introduces potential selection bias and residual confounding. Second, the sample size was limited, restricting statistical power, particularly for fracture outcomes, and limiting the robustness of multivariable, propensity-score-based, and sensitivity analyses. Third, baseline imbalances, including differences in follow-up duration, supplementation patterns, treatment adherence, and treatment discontinuation, may have influenced event detection and symptom trajectories. Radiological assessments were based on routine clinical imaging interpretation rather than standardized quantitative scoring systems, which may limit the reproducibility and interpretability of structural outcome evaluation. Because analgesic use, pain-management strategies, and visit timing were not standardized in this retrospective dataset, changes in VAS scores should also be interpreted cautiously and may partly reflect regression to the mean or routine-care heterogeneity rather than treatment exposure alone. The exploratory transcriptomic analyses incorporated independent GEO cohorts derived from different tissue sources, microarray platforms, and preprocessing conditions, which may have contributed to biological and technical heterogeneity across datasets. Propensity score matching was performed as a sensitivity analysis; however, given the limited number of matched pairs and incomplete covariate balance, these findings should be interpreted cautiously and were not considered robust for inference. Adverse-event and treatment-discontinuation data were available as part of routine clinical documentation and were therefore interpreted descriptively. Because the timing, severity, and causality of reported symptoms could not be standardized retrospectively, these observations were not used for formal comparative safety inference. Fourth, the molecular docking analyses were performed using static crystallographic protein structures under simplified computational conditions and therefore do not fully capture conformational dynamics, solvent effects, protonation variability, or the broader thermodynamic and kinetic complexity of ligand–protein interactions under physiological conditions. The exploratory multi-target docking framework evaluated potential structural interactions not only with the canonical pharmacological target FDPS but also with proteins associated with inflammation, bone remodeling, and nociceptive signaling. Consequently, potential binding tendencies observed outside the primary pharmacological pathway should not be considered evidence of functional inhibition or clinical effect in the absence of experimental validation.

Therefore, the present docking findings should be interpreted as supportive computational observations that may provide mechanistic context for the clinical and network-based analyses rather than as definitive evidence of biological activity.

## 5. Conclusions

In this retrospective exploratory study, ibandronate use was associated with numerically lower vertebral fracture incidence and greater unadjusted improvement in pain; however, these associations were attenuated after adjustment for baseline clinical characteristics. Radiological and symptomatic outcomes showed partial dissociation, suggesting that pain trajectories in osteoporosis are not solely determined by structural changes. These findings are compatible with the possibility of interplay between skeletal, inflammatory, and symptom-related pathways in osteoporosis. Given the limited sample size, the adjusted findings should be interpreted cautiously, and definitive conclusions regarding clinical effectiveness cannot be drawn. Prospective studies with larger sample sizes and integrated clinical and mechanistic approaches are warranted to further clarify these associations.

## Figures and Tables

**Table 1:** Baseline characteristics of the study population.

Variable	Ibandronate (*n* = 20)	Control (*n* = 20)	*p*-Value
Age (years)	63.65 ± 10.14	67.20 ± 8.64	0.241
BMI (kg/m^2^)	27.61 ± 4.32	27.15 ± 4.67	0.748
Menopause (years)	16.00 ± 7.00	16.60 ± 9.44	0.821
Lumbar BMD T-score, median (IQR)	−2.41 (−2.93 to −1.86)	−2.23 (−2.89 to −1.74)	0.766
Previous fracture, *n* (%)	12 (60%)	13 (65%)	1.000
Smoking, *n* (%)	6 (30%)	4 (20%)	0.716
Comorbidity, *n* (%)	14 (70%)	14 (70%)	1.000
Baseline VAS	6 (5–8)	5 (4–6)	0.071
Follow-up (months)	33 (17–45)	46 (28–59)	0.021
Ca/VitD use, *n* (%)	6 (30%)	14 (70%)	0.026

Data are presented as mean ± SD, median (IQR), or *n* (%), as appropriate.

**Table 2:** Association between ibandronate use and new vertebral fractures.

Model	OR	95% CI	*p*-Value
Crude	0.54	0.15–1.92	0.339
Adjusted *	0.37	0.08–1.67	0.197

* Adjusted for age, lumbar BMD T-score, and total follow-up duration. OR, odds ratio; CI, confidence interval.

**Table 3:** Adjusted linear regression for ΔVAS.

Variable	β	SE	95% CI	*p*-Value
Ibandronate	1.12	0.685	−0.27 to 2.51	0.110
Baseline VAS	1.26	0.192	0.87 to 1.65	<0.001
Age	−0.016	0.035	−0.09 to 0.06	0.655

β, unstandardized regression coefficient; SE, standard error; CI, confidence interval.

**Table 4:** Top enriched Gene Ontology (Biological Process and Molecular Function) terms and KEGG/Reactome pathways identified from the predefined target network using STRING enrichment analysis (false discovery rate-adjusted *p*-values).

Category	Term	Gene Count	FDR
GO BP	Ossification	13	6.06 × 10^−14^
	Positive regulation of acute inflammatory response	6	3.24 × 10^−9^
	Positive regulation of fever generation	5	3.24 × 10^−9^
	Response to lipopolysaccharide	11	3.32 × 10^−10^
	Regulation of inflammatory response	10	1.11 × 10^−8^
	Skeletal system development	11	9.24 × 10^−9^
GO MF	Cytokine activity	5	4.19 × 10^−2^
	Prostaglandin-endoperoxide synthase activity	2	4.19 × 10^−2^
KEGG Pathways	Osteoclast differentiation	10	6.63 × 10^−14^
	IL-17 signaling pathway	8	3.49 × 10^−11^
	TNF signaling pathway	8	1.05 × 10^−10^
	Rheumatoid arthritis	7	8.87 × 10^−10^
	Toll-like receptor signaling pathway	7	2.44 × 10^−9^
	C-type lectin receptor signaling pathway	7	2.44 × 10^−9^
Reactome Pathways	Cytokine Signaling in Immune system	11	5.03 × 10^−7^
	Signaling by Interleukins	8	5.07 × 10^−5^
	Interleukin-4 and Interleukin-13 signaling	5	1.20 × 10^−4^
	Interleukin-10 signaling	4	1.60 × 10^−4^

**Table 5:** Topological parameters of MCC-selected hub proteins.

Rank	Protein Symbol	Description	Degree	Betweenness Centrality	Closeness Centrality
1	IL6	Interleukin 6	9	0.042	1.000
2	TNF	Tumor necrosis factor	9	0.042	1.000
3	NFKB1	Nuclear factor NF-κB subunit 1	9	0.042	1.000
4	CTNNB1	Catenin beta-1	8	0.015	0.900
5	PTGS2	Prostaglandin-endoperoxide synthase 2	8	0.015	0.900
6	IL1B	Interleukin 1 beta	8	0.023	0.900
7	MAPK14	Mitogen-activated protein kinase 14	7	0.005	0.818
8	MMP9	Matrix metalloproteinase 9	7	0.011	0.818
9	MAPK1	Mitogen-activated protein kinase 1	6	0.000	0.750
10	TNFSF11	Tumor necrosis factor ligand superfamily member 11	5	0.000	0.692

**Table 6:** Top 10 hub proteins ranked by maximal clique centrality (MCC) using the cytoHubba plugin in Cytoscape. Raw MCC scores are presented as obtained from the analysis without transformation.

Rank	Protein Symbol	MCC Score
1	IL6	2298
2	TNF	2282
3	NFKB1	2280
4	CTNNB1	2170
5	PTGS2	2166
6	IL1B	1578
7	MAPK14	1440
8	MMP9	846
9	MAPK1	720
10	TNFSF11	168

**Table 7:** Disease enrichment analysis (DisGeNET).

Disease Term	Gene Overlap (k/N)	Adjusted *p*-Value	Representative Genes
Osteoporosis	17/441	1.08 × 10^−20^	*IL6*, *TNF*, *IL1B*, *TNFSF11*, *CTSK*
Osteopenia	17/329	1.15 × 10^−22^	*IL6*, *TNF*, *RUNX2*, *ALPL*
Bone diseases	15/165	6.33 × 10^−23^	*TNFSF11*, *CTSK*, *ACP5*
Osteoporosis (postmenopausal)	11/55	3.36 × 10^−20^	*IL6*, *TNF*, *NFKB1*
Rheumatoid arthritis	18/1833	1.85 × 10^−12^	*IL6*, *TNF*, *PTGS2*
Arthritis	18/630	4.20 × 10^−20^	*IL1B*, *TNF*, *MMP9*
Degenerative polyarthritis	23/976	4.31 × 10^−26^	*IL6*, *TNF*, *CTNNB1*
Pain/Hyperalgesia	8/119	~10^−11^	*IL6*, *TRPV1*, *PTGS2*

## Data Availability

The datasets generated and/or analyzed during the current study are available from the corresponding author on reasonable request. Additional supporting data are provided in the Supplementary Materials. Publicly available transcriptomic datasets analyzed in this study are accessible through the Gene Expression Omnibus (GEO) database under accession numbers GSE230665, GSE35958, and GSE7429.

## Supplementary Materials

The following supporting information can be downloaded at: https://www.mdpi.com/article/10.3390/biomedicines14061315/s1, Figure S1: Radiological progression and improvement by treatment group; Figure S2: Two-dimensional (2D) chemical structure of ibandronic acid; Figure S3: Gene Ontology molecular-function enrichment analysis of the selected target panel; Figure S4: Cytoscape-based network topology and hub-gene analysis; Figure S5: Disease enrichment analysis of the network-pharmacology target panel; Figure S6: Cross-cohort transcriptomic concordance landscape between GSE230665 and GSE35958; Figure S7: Hub-gene cross-cohort visualization across GEO transcriptomic datasets; Table S1: Extended baseline, laboratory, and additional clinical characteristics; Table S2: Full multivariable logistic regression model for new vertebral fracture; Table S3: Within-group changes in pain scores; Table S4: Propensity score matching and outcomes (sensitivity analysis); Table S5: Extended multivariable logistic regression for new vertebral fracture; Table S6: Extended multivariable linear regression for change in pain (ΔVAS); Table S7: Covariate balance before and after overlap weighting; Table S8: Overlap-weighted sensitivity analysis for clinical outcomes; Table S9: Molecular docking interaction profiles and binding parameters; Table S10: Shared differentially expressed genes between GSE230665 and GSE35958 with direction class; Table S11: Same-direction shared differentially expressed genes between GSE230665 and GSE35958; Table S12: Opposite-direction shared differentially expressed genes between GSE230665 and GSE35958; Table S13: Exploratory peripheral blood B-cell cohort (GSE7429) differential-expression output; Table S14: Cross-cohort status of the thirteen network-pharmacology hub genes.

## Abbreviations

The following abbreviations are used in this manuscript:

BMD	Bone mineral density
CI	Confidence interval
DEG	Differentially expressed gene
FDPS	Farnesyl pyrophosphate synthase
FDR	False discovery rate
GEO	Gene Expression Omnibus
GO	Gene Ontology
KEGG	Kyoto Encyclopedia of Genes and Genomes
MCC	Maximal clique centrality
OR	Odds ratio
PDB	Protein Data Bank
PMO	Postmenopausal osteoporosis
PPI	Protein–protein interaction
PSM	Propensity score matching
RMSD	Root mean square deviation
SD	Standard deviation
STRING	Search Tool for the Retrieval of Interacting Genes/Proteins
TNF	Tumor necrosis factor
VAS	Visual Analog Scale
VDR	Vitamin D receptor
