# Supplementary material for: Ibandronate Use in Osteoporotic Vertebral Fractures: A Retrospective Clinical Study Integrated with Exploratory Network Pharmacology and Cross-Cohort Transcriptomic Analysis
Source: Biomedicines. 2026 Jun 10;14(6):1315. doi: 10.3390/biomedicines14061315 (PMC13297545; doi:10.3390/biomedicines14061315)
Supplement: Supplementary file 1 [file biomedicines-14-01315-s001.zip › biomedicines-4357896-supplementary.pdf]

**Supplementary Materials**

Ibandronate Use in Osteoporotic Vertebral Fractures: A Retrospective Clinical Study Integrated with Exploratory Network Pharmacology and Cross-Cohort Transcriptomic Analysis

**Supplementary Note**

This supplementary file provides additional tables and figures supporting the clinical, transcriptomic, network-pharmacology, and computational analyses presented in the main manuscript. The supplementary materials include cross-dataset differential-expression analyses, hub-gene validation summaries, exploratory transcriptomic comparisons, and molecular docking results. All computational findings are presented within an exploratory and hypothesis-generating framework rather than as mechanistic confirmation.

**S1. Supplementary Methods**

***In Silico* Analysis Workflow Overview**

This supplementary document was prepared to provide a more detailed description of the methodological framework underlying the in silico analyses presented in the main manuscript. The primary aim of this section is not to expand the analytical scope of the study, but rather to enhance methodological transparency and improve the reproducibility of the computational workflow. Accordingly, the supplementary material presents the detailed molecular docking workflow, ligand and receptor preparation procedures, target-specific grid parameters, and residue-level interaction summaries derived from visualization analyses. The information provided herein is intended to support the interpretation of the main findings by documenting the technical aspects of the computational approach and facilitating a clearer understanding of the analytical procedures used throughout the study.

**Software and Computational Environment**

Molecular docking analyses were performed using AutoDock 4.2.6. Receptor and ligand preparation procedures, as well as grid box definitions, were conducted using AutoDockTools (MGLTools v1.5.6). Two-dimensional chemical structures of the ligands were initially generated using ChemDraw Ultra 12.0, followed by three-dimensional conformational modeling and energy minimization with the MMFF94 force field in ChemBio3D Ultra 13.0. Required file format conversions were subsequently performed using Open Babel (v3.1.1).

Following docking simulations, protein–ligand complexes were evaluated using BIOVIA Discovery Studio Visualizer (2025 Client) to assess binding orientations, residue-level interactions, and two- and three-dimensional interaction profiles. This computational framework was used to maintain methodological consistency throughout ligand preparation, docking simulations, interaction analysis, and structural visualization procedures.

**Ligand Preparation**

The chemical structures of the ligands used in this study were initially generated using ChemDraw Ultra 12.0. Following preparation of the two-dimensional structures, three-dimensional conformations

were generated in ChemBio3D Ultra 13.0 and subjected to energy minimization using the Merck Molecular Force Field (MMFF94). This procedure was performed to obtain energetically favorable and structurally stable conformations for each ligand. The minimized conformers were subsequently saved in protein data bank (PDB) format.

Ligand structures were then converted into the PDBQT format required for AutoDock analyses using Open Babel (v3.1.1). During this process, Gasteiger charges were assigned, polar hydrogen atoms were added, and rotatable bonds were defined according to AutoDock parameters. The number of rotatable bonds was adjusted while considering the structural characteristics and conformational flexibility of each ligand, with the aim of preserving sufficient molecular flexibility without unnecessarily expanding the conformational search space.

This preparation strategy was applied to facilitate a balanced evaluation of potential ligand–protein binding modes while maintaining computational efficiency and methodological consistency across different docking analyses. The resulting ligand files were subsequently used in a standardized manner throughout all molecular docking simulations performed in the study.

**Receptor Preparation**

The crystallographic structures of the target proteins used in this study were obtained from the Protein Data Bank (PDB) database. Receptor preparation procedures included the removal of crystallographic water molecules, deletion of co-crystallized ligands, addition of polar hydrogen atoms, and assignment of Kollman charges. All receptor structures were subsequently converted into the PDBQT format required for AutoDock analyses. Grid boxes were positioned to encompass the active binding regions, using the experimentally defined binding sites of co-crystallized ligands as reference points whenever available.

Grid box dimensions were standardized across all targets to maintain methodological comparability, whereas grid center coordinates were individually optimized according to the relevant binding region of each protein. This approach was intended to preserve target-specific biological relevance while also supporting comparative evaluation across different docking analyses.

**Docking Protocol**

Docking simulations were performed using the Lamarckian Genetic Algorithm (LGA) implemented in AutoDock 4.2.6. For each ligand–protein pair, forty independent docking runs were conducted. Grid maps were generated using AutoDockTools (MGLTools v1.5.6), and the grid spacing was set to 0.375 Å.

The resulting conformations were evaluated according to binding energy ($\Delta G$), root mean square deviation (RMSD), and cluster size criteria. Similar binding modes were clustered using an RMSD tolerance threshold of $\leq$ 2.0 Å. Conformations exhibiting the lowest binding energies within the most populated clusters were selected as representative binding modes for subsequent interaction analyses.

**Target Proteins and Grid Parameters**

Docking analyses were performed for the following target proteins using target-specific grid center coordinates and standardized grid dimensions. Grid parameters were selected to adequately encompass the relevant binding regions of each protein while maintaining methodological consistency across docking simulations.

FDPS (PDB ID: 1YV5)

Grid center: x = 15.167, y = 32.861, z = -8.917 Å
Grid size: 40 × 40 × 40 Å

CTSK (PDB ID: 1ATK)
Grid center: x = -18.139, y = -22.222, z = 59.139 Å
Grid size: 40 × 40 × 40 Å

MMP9 (PDB ID: 5I12)
Grid center: x = 19.778, y = -19.667, z = 19.806 Å
Grid size: 40 × 40 × 40 Å

COX-2 (PDB ID: 3LN1)
Grid center: x = 30.694, y = -22.444, z = -16.056 Å
Grid size: 40 × 40 × 40 Å

COX-1 (PDB ID: 3KK6)
Grid center: x = -32.167, y = 43.444, z = -6.139 Å
Grid size: 40 × 40 × 40 Å

TNF-$\alpha$ (PDB ID: 2AZ5)
Grid center: x = -18.611, y = 74.750, z = 33.222 Å
Grid size: 40 × 40 × 40 Å

IL-1β (PDB ID: 5R85)
Grid center: x = 39.194, y = 3.833, z = 73.389 Å
Grid size: 40 × 40 × 40 Å

MAPK14 (PDB ID: 1A9U)
Grid center: x = 3.361, y = 15.278, z = 28.278 Å
Grid size: 40 × 40 × 40 Å

VDR (PDB ID: 1DB1)
Grid center: x = 10.639, y = 22.083, z = 34.361 Å
Grid size: 40 × 40 × 40 Å

MAPK1 (PDB ID: 2OJJ)

Grid center: x = -13.889, y = 13.806, z = 40.917 Å
Grid size: 40 × 40 × 40 Å

NF-κB (PDB ID: 1LE9)
Grid center: x = 117.674, y = 18.111, z = 19.444 Å
Grid size: 40 × 40 × 40 Å

ACP5 (PDB ID: 2BQ8)
Grid center: x = 2.361, y = 55.417, z = 46.944 Å
Grid size: 40 × 40 × 40 Å

PTH1R (PDB ID: 6FJ3)
Grid center: x = 52.000, y = -109.000, z = 206.000 Å
Grid size: 40 × 40 × 40 Å

SCN9A (PDB ID: 7XMF)
Grid center: x = 134.833, y = 131.389, z = 132.889 Å
Grid size: 40 × 40 × 40 Å

**Interaction Analysis**

Protein–ligand complexes obtained from docking simulations were transferred to BIOVIA Discovery Studio Visualizer for detailed interaction assessment. For each complex, ligand positioning within the target protein, binding orientation, and interactions with relevant amino acid residues were examined. Analyses particularly focused on conventional hydrogen bonds, attractive charge interactions, alkyl/pi-alkyl contacts, and pi-sigma interactions.

In addition to binding energy values, the structural positioning of ligands within the corresponding binding regions and their associated non-covalent interaction patterns were also considered during the evaluation process. Two-dimensional (2D) interaction diagrams generated for each target were used to visualize residue-level contacts between ligands and amino acid residues, whereas three-dimensional (3D) complex structures were examined to assess the spatial orientation of ligands within the binding pocket. This approach was intended to support structural interpretation of the docking findings beyond numerical scoring parameters alone.

**Methodological Considerations**

The molecular docking approach used in this study enables structural evaluation of potential ligand–protein interactions; however, several methodological limitations should be considered. Docking simulations were performed using static crystallographic structures and therefore may not fully capture the dynamic conformational behavior of proteins under physiological conditions. In addition, docking scoring functions provide approximate estimates of binding affinity and do not fully represent the thermodynamic and biological complexity of protein–ligand interactions. Accordingly, the present findings should be interpreted as exploratory computational observations rather than direct evidence of biological activity or mechanistic validation. Further experimental studies and molecular dynamics simulations would be required for biological confirmation.

## S2. Supplementary Results

### S2.1. Clinical Outcomes

**Table S1.** Extended baseline, laboratory, and additional clinical characteristics

| Variable | Ibandronate (n=20) | Control (n=20) | p-value |
|---|---|---|---|
| Height, cm | 159.00 (155.75–166.25) | 164.00 (157.25–166.00) | 0.524 |
| Weight, kg | 75.50 (60.00–79.00) | 73.00 (62.00–79.75) | 0.892 |
| Femoral neck BMD T-score | -2.06 ± 0.48 | -2.10 ± 0.47 | 0.787 |
| Total hip BMD T-score | -2.33 (-2.69 to -2.03) | -2.05 (-2.54 to -1.48) | 0.144 |

| | | | |
|---|---|---|---|
| 25(OH) vitamin D | 17.00 (14.00–33.50) | 30.50 (19.75–35.00) | 0.217 |
| Calcium | 9.35 ± 0.66 | 9.48 ± 0.53 | 0.503 |
| Phosphorus | 3.58 ± 0.57 | 3.43 ± 0.50 | 0.381 |
| ALP | 90.50 (73.25–118.75) | 83.50 (73.75–118.75) | 0.829 |
| PTH | 43.25 ± 15.29 | 51.80 ± 18.73 | 0.122 |
| Fracture count | 0.50 (0.00–1.25) | 0.50 (0.00–2.00) | 0.641 |
| Non-traumatic fracture, n (%) | 9 (45%) | 10 (50%) | 1.000 |
| Regular adherence, n (%) | 14 (70%) | 11 (55%) | 0.514 |
| Initial deformity, n (%) | 13 (65%) | 14 (70%) | 1.000 |
| Bone pain, n (%) | 11 (55%) | 7 (35%) | 0.341 |
| Musculoskeletal pain, n (%) | 5 (25%) | 10 (50%) | 0.191 |
| Mobility status, n (%) | | | 0.765 |
| Assisted | 8 (40%) | 6 (30%) | |
| Independent | 7 (35%) | 9 (45%) | |
| Limited | 5 (25%) | 5 (25%) | |
| Any side effect, n (%) | 17 (85%) | 0 (0%) | <0.001 |
| Any treatment discontinuation reason, n (%) | 12 (60%) | 0 (0%) | <0.001 |

Continuous variables are presented as mean ± SD or median (IQR), according to distribution. Categorical variables are presented as n (%).

**Table S2.** Full multivariable logistic regression model for new vertebral fracture

| Variable | OR | 95% CI | p-value |
|---|---|---|---|
| Ibandronate group | 0.37 | 0.08–1.67 | 0.197 |
| Age | 1.03 | 0.96–1.12 | 0.411 |
| Lumbar spine BMD T-score | 1.39 | 0.44–4.40 | 0.574 |
| Total follow-up duration, months | 0.96 | 0.91–1.01 | 0.088 |

Model note: Adjusted logistic model = New_Fracture ~ Group + Age + BMD_Lumbar_T + Total_Followup_months.

**Table S3.** Within-group changes in pain scores

| Group | Baseline VAS | Follow-up VAS | ΔVAS | Paired t-test p-value | Wilcoxon p-value |
|---|---|---|---|---|---|
| Ibandronate | 6.45 ± 1.85 | 4.80 ± 2.19 | 1.65 ± 2.91 | 0.020 | 0.025 |
| Control | 5.40 ± 1.57 | 6.25 ± 1.83 | -0.85 ± 2.92 | 0.209 | 0.153 |

ΔVAS = Baseline_VAS - Followup_VAS; positive values indicate pain reduction.

**Table S4.** Propensity score matching and outcomes (sensitivity analysis)

| Variable / Outcome | Before Matching (SMD) | After Matching (SMD) | Ibandronate (n=6) | Control (n=6) | p-value |
|---|---|---|---|---|---|
| Age | -0.377 | -0.143 | NA | NA | NA |
| BMI | 0.102 | -0.055 | NA | NA | NA |
| Smoking | 0.205 | 0.358 | NA | NA | NA |
| Comorbidity | 0.214 | 0.690 | NA | NA | NA |
| Lumbar BMD T-score | -0.103 | 0.712 | NA | NA | NA |
| Previous fracture | 0.000 | 0.313 | NA | NA | NA |
| Baseline VAS | 0.612 | 0.160 | NA | NA | NA |
| Ca/VitD use | -0.851 | 0.000 | NA | NA | NA |
| New fracture, n (%) | NA | NA | 3 (50%) | 4 (66.7%) | 1.000* |
| ΔVAS | NA | NA | 1.17 ± 2.71 | -1.17 ± 3.71 | 0.268† |

NA: Not applicable, * Exact McNemar test, † Paired t-test across matched pairs

Propensity score matching yielded a limited number of matched pairs (n = 6). Although balance improved for some covariates, residual imbalance persisted for others; therefore, these findings should be interpreted as exploratory sensitivity analyses.

**Table S5.** Extended multivariable logistic regression for new vertebral fracture (sensitivity analysis)

| Variable | OR | 95% CI | p-value |
|---|---|---|---|
| Ibandronate (yes vs no) | 0.50 | 0.10–2.53 | 0.405 |
| Age | 1.03 | 0.95–1.13 | 0.430 |
| Lumbar spine BMD T-score | 1.41 | 0.43–4.59 | 0.561 |

| | | | |
|---|---|---|---|
| Total follow-up duration (months) | 0.96 | 0.91–1.02 | 0.105 |
| Ca/VitD use (yes vs no) | 0.71 | 0.15–3.42 | 0.660 |

**Model note:** Adjusted logistic model = New_Fracture ~ Group + Age + Lumbar_BMD_T + Follow-up + Ca/VitD

**Table S6.** Extended multivariable linear regression for change in pain (ΔVAS)

| Variable | β | SE | 95% CI | p-value |
|---|---|---|---|---|
| Ibandronate (yes vs no) | 1.18 | 0.78 | −0.39 to 2.76 | 0.136 |
| Baseline VAS | 1.24 | 0.20 | 0.83 to 1.64 | <0.001 |
| Age | −0.02 | 0.04 | −0.10 to 0.06 | 0.610 |
| Total follow-up duration (months) | −0.01 | 0.02 | −0.04 to 0.03 | 0.550 |
| Ca/VitD use (yes vs no) | 0.48 | 0.85 | −1.24 to 2.21 | 0.570 |

**Table S7.** Covariate balance before and after overlap weighting

| Variable | Before weighting (SMD) | After weighting (SMD) |
|---|---|---|
| Age | −0.377 | −0.028 |
| BMI | 0.102 | 0.012 |
| Smoking | 0.205 | 0.041 |
| Comorbidity | 0.214 | 0.036 |
| Lumbar BMD T-score | −0.103 | −0.019 |
| Previous fracture | 0.000 | 0.000 |
| Baseline VAS | 0.612 | 0.045 |
| Ca/VitD use | −0.851 | −0.022 |

Abbreviation: SMD, standardized mean difference

**Table S8.** Overlap-weighted sensitivity analysis for clinical outcomes

| Outcome | Variable | Effect Estimate | 95% CI | p-value |
|---|---|---|---|---|
| ΔVAS (weighted linear regression) | Ibandronate (yes vs no) | β = 1.22 | −0.06 to 2.49 | 0.062 |
| New vertebral fracture (weighted logistic regression) | Ibandronate (yes vs no) | OR = 0.48 | 0.12–1.98 | 0.310 |

Abbreviations: ΔVAS, change in visual analog scale; β, unstandardized regression coefficient; OR, odds ratio; CI, confidence interval.

**Model note:** Overlap-weighted models were constructed using propensity scores estimated from baseline covariates (age, body mass index, smoking status, comorbidity, lumbar spine bone mineral density T-score, previous fracture, baseline VAS, and calcium/vitamin D use).

### S2.2. Molecular Docking Results

Detailed docking scores and interaction profiles are provided in the supplementary tables.

**Table S9.** Molecular docking results of ibandronic acid against the selected target proteins, including binding energies, RMSD values, and non-covalent interaction profiles.

| Compound | Binding energy (kcal/mol) | RMSD | Conventional Hydrogen Bond | Attractive Charge | Alkyl/pi-Alkyl | Pi-sigma | Target protein | PDB ID |
|---|---|---|---|---|---|---|---|---|
| Ibandronic acid | -7.36 | 0.67 | Lys214Tyr218Gln254 | Asp117 | - | - | FDPS | 1YV5 |
| Ibandronic acid | -6.74 | 0.94 | Cys25Gly66Met68 | - | Tyr67 | - | CTSK | 1ATK |
| Ibandronic acid | -10.03 | 1.13 | Ala189Leu188Glu227 | - | Tyr248 | His226 | MMP9 | 5I12 |
| Ibandronic acid | -8.35 | 0.94 | Ser516Gly512Ala513 | - | Val509Phe504Leu338Ala502 | - | COX-2 | 3LN1 |
| Ibandronic acid | -8.07 | 1.05 | Tyr385Gly526Ser530 | - | Val349Ala527 | - | COX-1 | 3KK6 |
| Ibandronic acid | -5.39 | 1.33 | Tyr151Ser60Gln61 | - | Tyr119 | - | TNF-α | 2AZ5 |
| Ibandronic acid | -8.96 | 0.98 | Tyr24Leu26 | Glu25 | Lys77 | - | IL-1β | 5R85 |
| Ibandronic acid | -5.81 | 0.83 | Lys53Asp168 | Asp168Glu71 | Ala51Leu108 | - | MAPK14 | 1A9U |
| Ibandronic acid | -8.29 | 1.19 | Ser278Ile271 | - | Tyr295Val234Leu233Trp286 | Trp286 | VDR | 1DB1 |
| Ibandronic acid | -7.28 | 0.90 | Asp104Met106 | - | Leu154Ile29 | - | MAPK1 | 2OJJ |
| Ibandronic acid | -5.45 | 0.64 | Arg33Phe34Arg35 | - | Arg41Val91 | - | NF-κB | 1LE9 |
| Ibandronic acid | -6.92 | 1.00 | Arg79Arg108Lys107 | Glu70 | Ile105 | - | ACP5 | 2BQ8 |
| Ibandronic acid | -7.79 | 0.46 | Met425Lys360Tyr429Ser355 | - | Tyr429 | - | PTH1R | 6FJ3 |
| Ibandronic acid | -6.85 | 1.07 | Asn395Val1751 | - | Val1751Ile1744 | - | SCN9A | 7XMF |

## S3. Supplementary Figures

### S3.1. Radiological outcomes

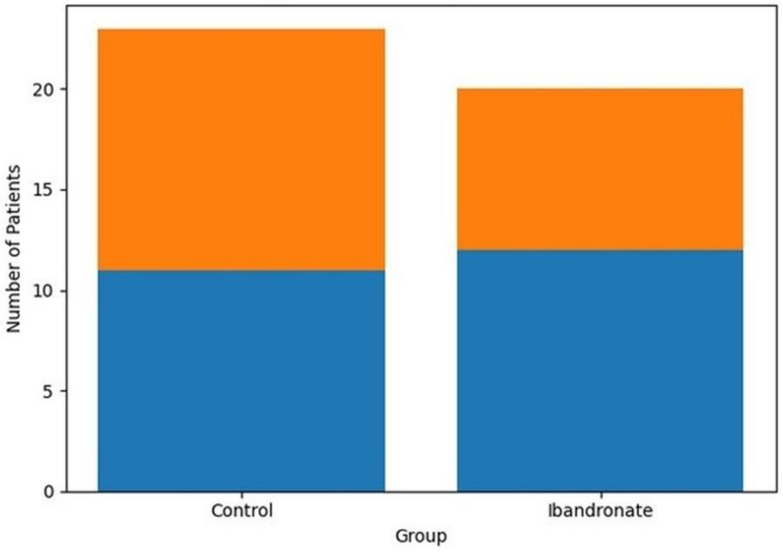


**Figure S1.** Radiological progression and improvement by treatment group. Stacked bar chart showing the distribution of radiological progression and radiological improvement in the ibandronate and control groups during follow-up. Data are presented as number of patients.

### S3.2. Molecular Docking Results

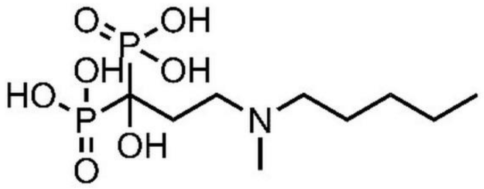


**Figure S2.** Two-dimensional (2D) chemical structure of ibandronic acid used in the molecular docking analyses. The structural backbone and major functional groups of the molecule are illustrated.

### S3.3. Network and Functional Enrichment Analyses

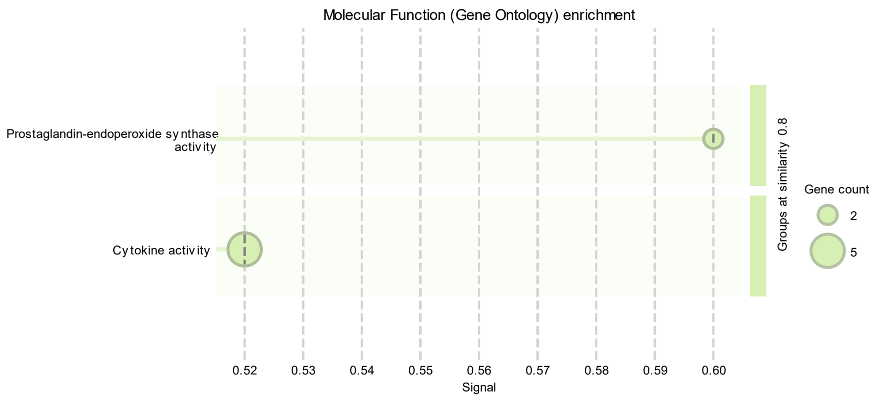


**Figure S3.** Molecular Function (Gene Ontology) enrichment analysis of the selected target panel. Exploratory analysis showed enrichment in cytokine activity and prostaglandin-endoperoxide synthase activity. Bubble size indicates gene count and color intensity reflects FDR-adjusted significance levels.

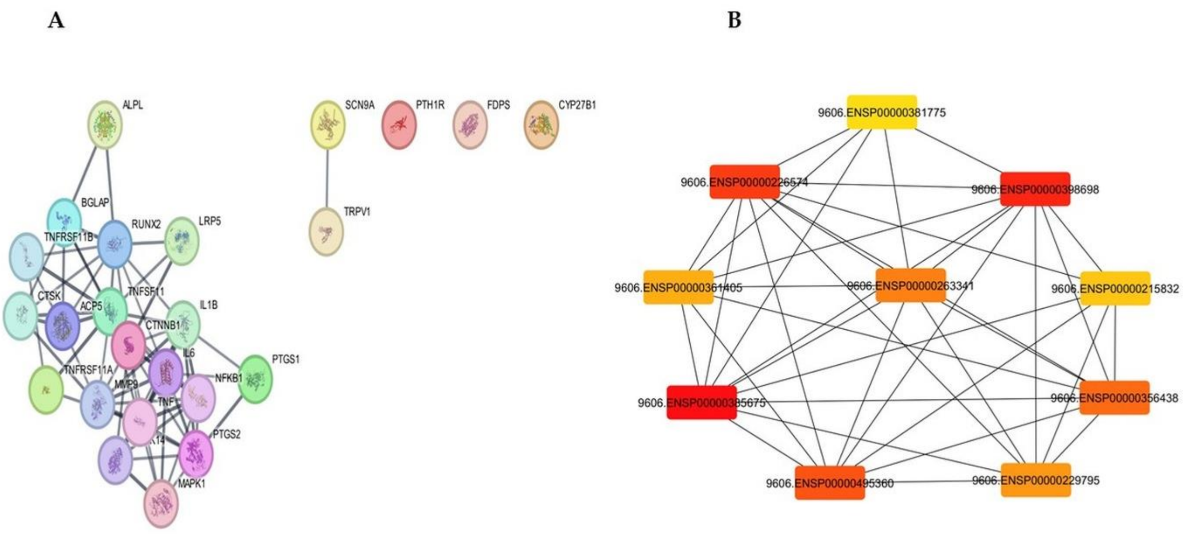


**Figure S4.** Cytoscape-based network topology and hub gene analysis. (A) The full protein–protein interaction network visualized in Cytoscape (version 3.10.4), where node size reflects degree centrality. (B) Subnetwork of the top 10 hub genes identified using the cytoHubba plugin based on the maximal clique centrality (MCC) algorithm. Nodes with higher MCC scores are highlighted, indicating relatively central positions within the network.

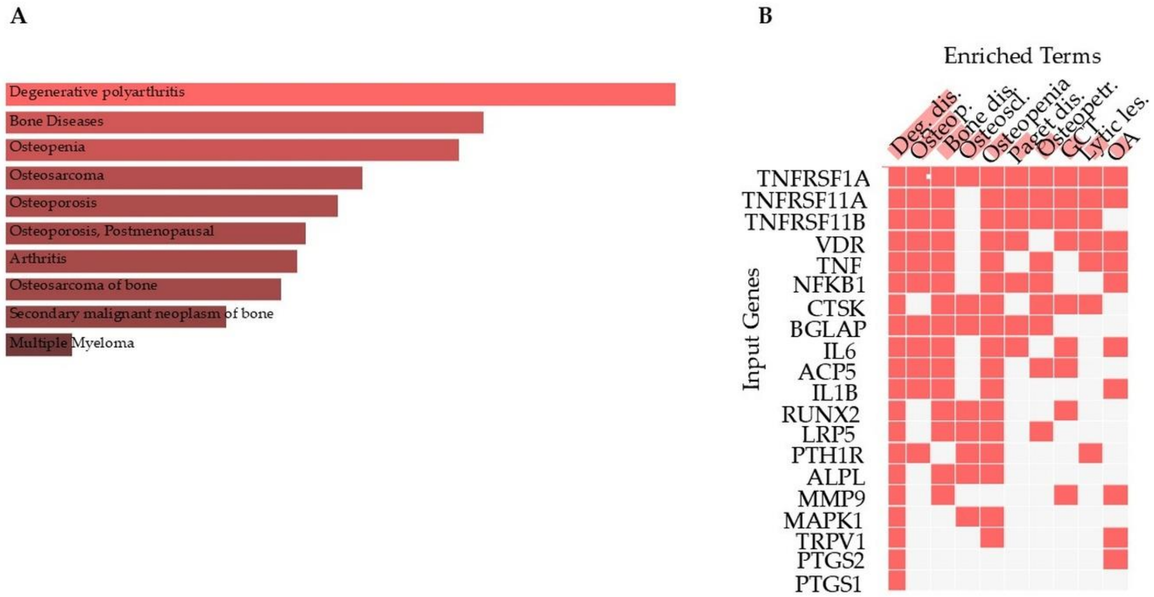


**Figure S5.** Disease enrichment analysis of the network-pharmacology target panel based on the DisGeNET database. (A) Top enriched disease terms identified from the network-pharmacology target panel. (B) Heatmap showing the associations between input genes and enriched disease categories. Abbreviations: Deg. dis., degenerative diseases; Bone dis., bone diseases; Osteop., osteoporosis; Osteoscl., osteosclerosis; Paget dis., Paget disease of bone; Osteopetr., osteopetrosis; GCT, giant cell tumor; Lytic les., lytic lesions; OA, osteoarthritis. Red indicates the presence of an association between the gene and the disease term, whereas grey indicates no reported association within the analyzed dataset.

### S3.4. Transcriptomic Analyses Across Independent GEO Cohorts

### S4. Exploratory peripheral blood B-cell cohort (GSE7429)

Under the same unified DEG threshold (|log2 fold-change| ≥ 1.0 and adj.P.Val < 0.05) and the high-BMD − low-BMD contrast applied identically to GSE7429, the peripheral B-cell cohort showed limited concordance with the tissue-derived PMO signatures derived from GSE230665 and GSE35958. Transcriptomic separation between the two BMD strata in GSE7429 was weak under stringent criteria, and the network-pharmacology hub-gene panel did not reach the DEG threshold in this cohort. We interpret this limited concordance cautiously and primarily as a reflection of tissue and cell-type heterogeneity — circulating B cells versus bone tissue and bone-marrow-derived mesenchymal stem/stromal cells. Consistent with this view, GSE7429 was retained in the analysis as a transparency comparator and as a negative/orthogonal control, and is reported in Supplementary Materials (Supplementary Figure S6, Supplementary Figure S7, and Supplementary Table S13). It is not interpreted as evidence against the network-pharmacology framework.

In summary, the supplementary peripheral blood B-cell analysis indicates that the bone-context transcriptomic signal recovered in the discovery and validation cohorts is not directly recapitulated in circulating B lymphocytes under matched stringent thresholds. This observation underscores the tissue-specific nature of the PMO transcriptomic signatures examined here and supports a hypothesis-generating, rather than mechanistic, interpretation of the cross-cohort findings.

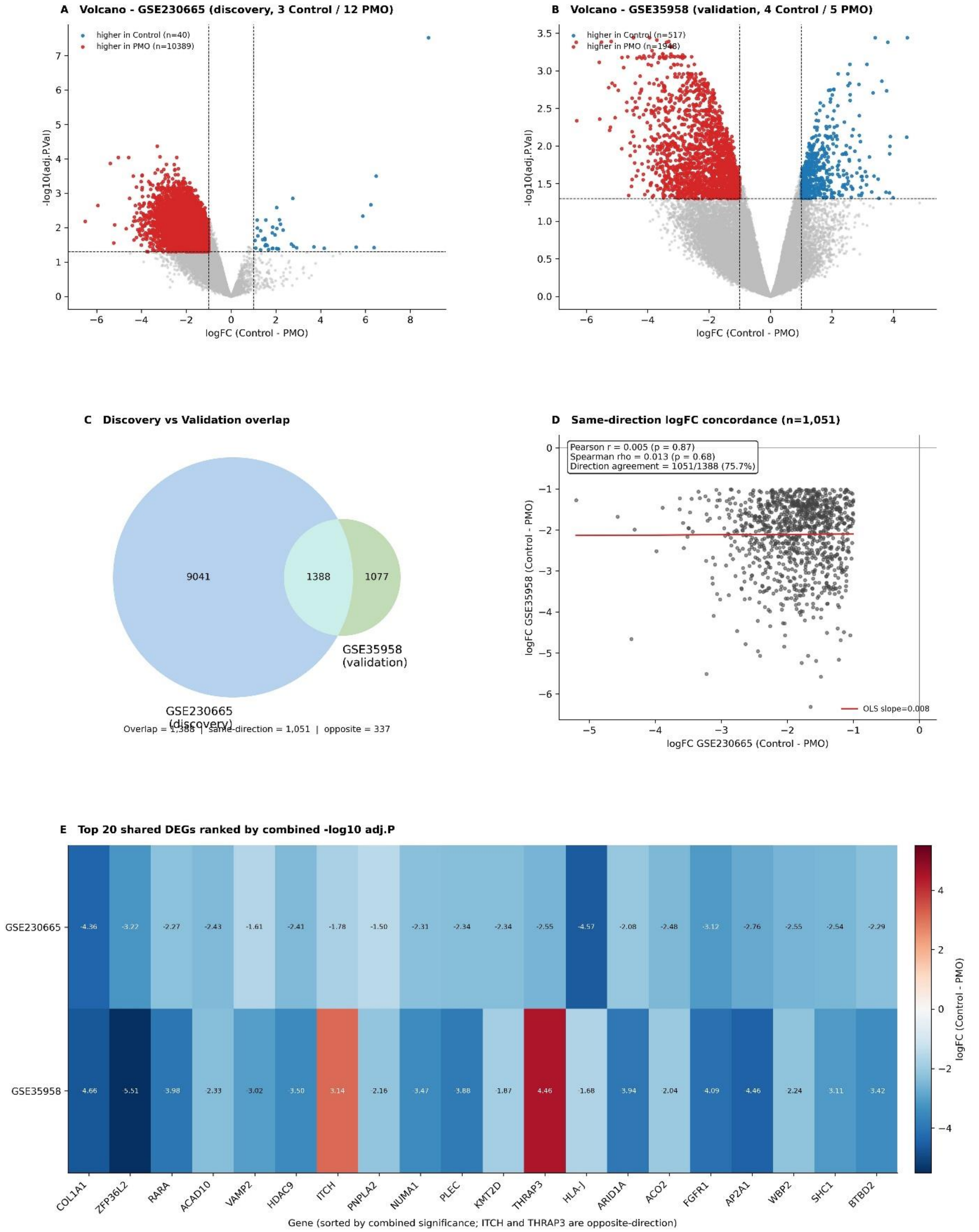


**Figure S6.** Cross-cohort transcriptomic concordance landscape of differentially expressed genes between the discovery dataset (GSE230665) and the independent comparison dataset (GSE35958). Cohort-specific differential-expression distributions and the concordant overlap of shared DEGs are presented under a harmonized analytical framework using the same Control − PMO contrast definition and unified significance criteria (|log2 fold-change| ≥ 1.0 and adjusted P value < 0.05). The figure summarizes the degree of transcriptomic concordance and directional consistency across independent GEO cohorts.

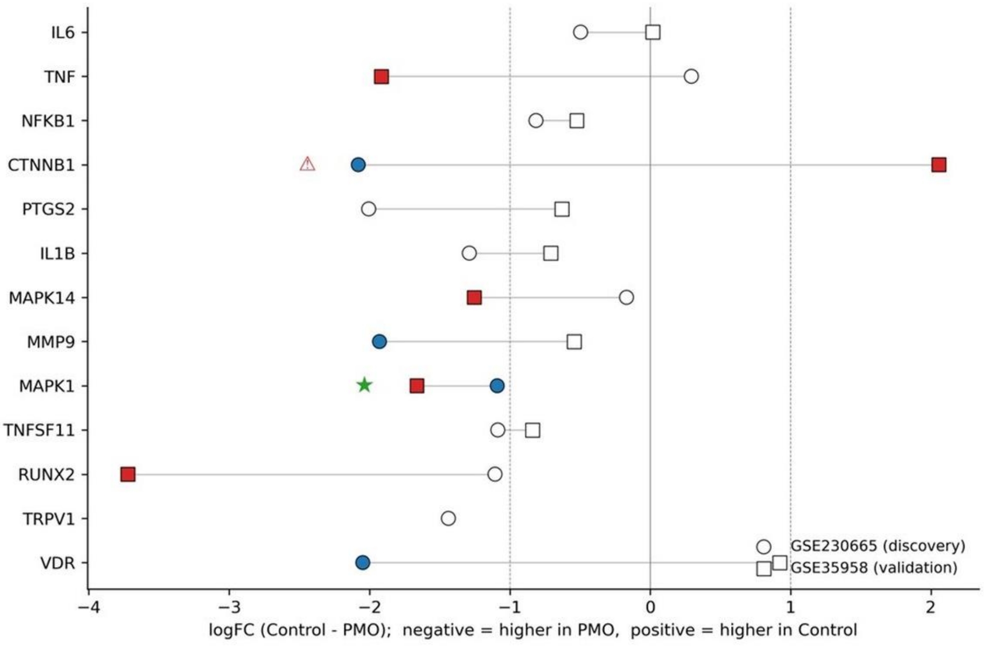


**Figure S7.** Hub-gene cross-cohort visualization across GEO transcriptomic datasets. Per-cohort log2 fold-change values are shown for the thirteen network-pharmacology hub genes (MAPK1, CTNNB1, MMP9, VDR, TNF, MAPK14, RUNX2, IL6, IL1B, NFKB1, PTGS2, TNFSF11, and TRPV1) under the unified Control − PMO contrast in GSE230665 and GSE35958, together with the high-BMD − low-BMD contrast in GSE7429. Filled markers denote genes meeting the predefined differential expression threshold (|logFC| ≥ 1.0 and adjusted P value < 0.05) within the corresponding dataset. Circular markers represent GSE230665, square markers represent GSE35958, and triangular markers represent GSE7429. Red color coding indicates relatively higher expression in the second comparator group, blue indicates relatively higher expression in the first comparator group, and grey denotes genes not detected or not meeting significance criteria in the corresponding dataset.

**Supplementary tables (cross-cohort transcriptomic comparison)**

**Table S10.** Complete list of shared differentially expressed genes between GSE230665 and GSE35958 (n = 1,388) with direction class. All gene symbols meeting the unified DEG threshold (|log2 fold-change| ≥ 1.0 and adj.P.Val < 0.05) in both the discovery cohort (GSE230665) and the independent comparison cohort (GSE35958) are shown. Each row reports the per-cohort log2 fold-change and adjusted p-value under the Control − PMO sign convention, together with a direction class (same-direction, n = 1,051; opposite-direction, n = 337). Genes are ordered according to combined statistical significance across the two cohorts.

| Gene Symbol | logFC GSE230665 | adj.P.Val GSE230665 | logFC GSE35958 | adj.P.Val GSE35958 | Direction Class |
|---|---|---|---|---|---|
| A4GALT | -1.953 | 0.00905 | -1.807 | 0.04572 | same direction |
| AAAS | -1.98 | 0.00892 | -1.938 | 0.01251 | same direction |
| ABCA5 | -1.688 | 0.00571 | 1.592 | 0.02901 | opposite direction |
| ABCB8 | -1.892 | 0.0152 | -3.442 | 0.02532 | same direction |
| ABCC1 | -2.772 | 0.0326 | -2.009 | 0.007938 | same direction |
| ABCD1 | -1.727 | 0.0128 | -1.42 | 0.01906 | same direction |
| ABHD12 | -1.491 | 0.0189 | -1.474 | 0.007302 | same direction |
| ABHD14B | -1.615 | 0.0339 | -1.988 | 0.002928 | same direction |

| | | | | | |
|---|---|---|---|---|---|
| ABHD6 | -2.292 | 0.00906 | -1.383 | 0.0145 | same direction |
| ABHD8 | -2.141 | 0.00197 | -1.743 | 0.01725 | same direction |
| ABI2 | -1.615 | 0.0133 | -1.354 | 0.01504 | same direction |
| ABR | -2.069 | 0.00312 | -2.321 | 0.006148 | same direction |
| ABTB1 | -1.221 | 0.00499 | -5.161 | 0.001455 | same direction |
| ACAD10 | -2.429 | 9.06e-05 | -2.333 | 0.009937 | same direction |
| ACADSB | -1.5 | 0.00728 | 1.258 | 0.04852 | opposite direction |
| ACBD4 | -1.808 | 0.00212 | -1.948 | 0.005616 | same direction |
| ACER3 | -1.987 | 0.00961 | 1.273 | 0.02921 | opposite direction |
| ACO2 | -2.481 | 0.00213 | -2.043 | 0.001505 | same direction |
| ACOT8 | -1.425 | 0.0201 | -1.102 | 0.02133 | same direction |
| ACTB | -1.957 | 0.00499 | -1.015 | 0.03866 | same direction |

**Table S11.** Complete list of same-direction shared differentially expressed genes between GSE230665 and GSE35958 (n = 1,051). All gene symbols meeting the unified DEG threshold (|log2 fold-change| ≥ 1.0 and adj.P.Val < 0.05) in both the discovery cohort (GSE230665) and the validation cohort (GSE35958) and showing concordant expression direction under the Control − PMO sign convention are shown. Each row reports the per-cohort log2 fold-change and adjusted p-values for the two cohorts. Genes are ordered by combined statistical significance across the two cohorts.

| Gene Symbol | logFC GSE230665 | adj.P.Val GSE230665 | logFC GSE35958 | adj.P.Val GSE35958 | Direction Class |
|---|---|---|---|---|---|
| A4GALT | -1.953 | 0.00905 | -1.807 | 0.04572 | same direction |
| AAAS | -1.98 | 0.00892 | -1.938 | 0.01251 | same direction |
| ABCB8 | -1.892 | 0.0152 | -3.442 | 0.02532 | same direction |
| ABCC1 | -2.772 | 0.0326 | -2.009 | 0.007938 | same direction |
| ABCD1 | -1.727 | 0.0128 | -1.42 | 0.01906 | same direction |
| ABHD12 | -1.491 | 0.0189 | -1.474 | 0.007302 | same direction |
| ABHD14B | -1.615 | 0.0339 | -1.988 | 0.002928 | same direction |
| ABHD6 | -2.292 | 0.00906 | -1.383 | 0.0145 | same direction |
| ABHD8 | -2.141 | 0.00197 | -1.743 | 0.01725 | same direction |
| ABI2 | -1.615 | 0.0133 | -1.354 | 0.01504 | same direction |
| ABR | -2.069 | 0.00312 | -2.321 | 0.006148 | same direction |
| ABTB1 | -1.221 | 0.00499 | -5.161 | 0.001455 | same direction |
| ACAD10 | -2.429 | 9.06e-05 | -2.333 | 0.009937 | same direction |
| ACBD4 | -1.808 | 0.00212 | -1.948 | 0.005616 | same direction |
| ACO2 | -2.481 | 0.00213 | -2.043 | 0.001505 | same direction |
| ACOT8 | -1.425 | 0.0201 | -1.102 | 0.02133 | same direction |
| ACTB | -1.957 | 0.00499 | -1.015 | 0.03866 | same direction |
| ACTN4 | -2.551 | 0.0113 | -1.901 | 0.02484 | same direction |
| ACTR1B | -1.686 | 0.0206 | -1.11 | 0.04392 | same direction |
| ADAM15 | -1.664 | 0.0201 | -2.155 | 0.002144 | same direction |

**Table S12.** Opposite-direction shared differentially expressed genes between GSE230665 and GSE35958 (n = 337). Subset of Supplementary Table S10 comprising genes with discordant log2 fold-change direction between the two cohorts under the Control − PMO sign convention. These genes are reported transparently without reconciliation into a unified expression direction to support cautious interpretation of the discovery–validation overlap. Each row reports the per-cohort log2 fold-change

and adjusted p-values for the two cohorts. Genes are ordered by combined statistical significance across the two cohorts.

| Gene Symbol | logFC GSE230665 | adj.P.Val GSE230665 | logFC GSE35958 | adj.P.Val GSE35958 | **D**irection **C**lass |
|---|---|---|---|---|---|
| ABCA5 | -1.688 | 0.00571 | 1.592 | 0.02901 | opposite direction |
| ACADSB | -1.5 | 0.00728 | 1.258 | 0.04852 | opposite direction |
| ACER3 | -1.987 | 0.00961 | 1.273 | 0.02921 | opposite direction |
| ADAM12 | -1.462 | 0.0124 | 2.104 | 0.007483 | opposite direction |
| AFG3L2 | -1.841 | 0.0297 | 1.105 | 0.02524 | opposite direction |
| AGK | -1.708 | 0.0342 | 1.1 | 0.03618 | opposite direction |
| AGTPBP1 | -1.545 | 0.00449 | 2.34 | 0.0128 | opposite direction |
| ALG10B | -2.001 | 0.00603 | 1.141 | 0.03551 | opposite direction |
| ANK3 | -2.385 | 0.00659 | 1.829 | 0.02143 | opposite direction |
| ANKRD11 | -1.853 | 0.0351 | 3.781 | 0.001839 | opposite direction |
| ANKRD12 | -3.376 | 0.00442 | 4.435 | 0.007604 | opposite direction |
| ANKRD16 | -1.52 | 0.0106 | 1.792 | 0.04877 | opposite direction |
| ANKRD36B | -1.716 | 0.00695 | 1.97 | 0.0299 | opposite direction |
| ANOS1 | -2.796 | 0.00483 | 2.247 | 0.02545 | opposite direction |
| ANP32A | -1.915 | 0.0169 | 1.383 | 0.02792 | opposite direction |
| APP | -2.66 | 0.0173 | 2.404 | 0.00479 | opposite direction |
| ARGLU1 | -2.497 | 0.0169 | 2.061 | 0.001752 | opposite direction |
| ARHGAP20 | -1.772 | 0.012 | 1.721 | 0.03313 | opposite direction |
| ARHGAP32 | -1.938 | 0.0277 | 2.377 | 0.0242 | opposite direction |
| ARID1B | -1.594 | 0.0131 | 1.122 | 0.02121 | opposite direction |

**Table S13.** Exploratory peripheral blood B-cell cohort (GSE7429): full gene-level differential expression output. Complete probe-to-symbol-collapsed gene-level differential expression statistics for GSE7429 under the high-BMD − low-BMD contrast, including log2 fold-change and adjusted p-values for all annotated gene symbols. This dataset was included as an exploratory peripheral blood B-cell comparator to the tissue-derived discovery and validation cohorts and was not interpreted as confirmatory validation.

| Gene Symbol | log2FC | t | P value | adj_P value | B |
|---|---|---|---|---|---|
| PTOV1-AS2 | 0.2552 | 5.984 | 6.27e-06 | 0.14 | 2.288 |
| STK11 | 0.2514 | 5.277 | 3.18e-05 | 0.275 | 1.275 |
| MAPK3 | 0.2188 | 5.212 | 3.697e-05 | 0.275 | 1.178 |
| MMP23A | 0.1736 | 4.745 | 0.0001111 | 0.475 | 0.4596 |
| HAO2 | 0.1831 | 4.686 | 0.000128 | 0.475 | 0.3657 |
| TMEM8B | 0.2032 | 4.503 | 0.0001974 | 0.572 | 0.076 |
| PSTPIP1 | 0.3208 | 4.487 | 0.0002053 | 0.572 | 0.0495 |
| UBQLN4 | 0.3505 | 3.72 | 0.001276 | 0.573 | -1.203 |
| DOHH | 0.3073 | 3.899 | 0.0008343 | 0.573 | -0.908 |
| MED13L | 0.2033 | 3.931 | 0.000773 | 0.573 | -0.8552 |
| ARPC4-TTLL3 | 0.2244 | 3.934 | 0.0007666 | 0.573 | -0.8495 |
| PART1 | 0.2275 | 3.942 | 0.0007531 | 0.573 | -0.8373 |
| BCAT2 | 0.2765 | 3.99 | 0.0006716 | 0.573 | -0.7582 |
| ESR1 | 0.1963 | 3.982 | 0.0006842 | 0.573 | -0.7709 |
| TAOK2 | 0.2663 | 3.882 | 0.0008688 | 0.573 | -0.9361 |
| ARMCX4 | 0.1768 | 3.99 | 0.0006713 | 0.573 | -0.7578 |

| | | | | | |
|---|---|---|---|---|---|
| ZNF446 | 0.1755 | 4.147 | 0.0004614 | 0.573 | -0.5003 |
| ACKR1 | 0.1678 | 3.959 | 0.0007224 | 0.573 | -0.8085 |
| PRG3 | 0.1543 | 3.876 | 0.0008812 | 0.573 | -0.9459 |
| SULT1A2 | 0.2915 | 3.836 | 0.0009682 | 0.573 | -1.011 |

**Table S14.** Cross-cohort status of the thirteen network-pharmacology hub genes. For each of the thirteen hub genes (MAPK1, CTNNB1, MMP9, VDR, TNF, MAPK14, RUNX2, IL6, IL1B, NFKB1, PTGS2, TNFSF11, and TRPV1), the per-cohort log2 fold-change, adjusted p-value, and DEG status under the unified threshold (|log2 fold-change| ≥ 1.0 and adj.P.Val < 0.05) are reported separately for GSE230665 and GSE35958. A pre-specified cross-cohort classification is also provided for each gene: cross-validated, discordant, discovery-only, validation-only, not differentially expressed, or absent on platform. MAPK1 was the only same-direction cross-validated hub gene, CTNNB1 was classified as discordant, and TRPV1 was not represented on the GSE35958 platform.

| **Hub gene** | **GSE230665 logFC** | **GSE230665 adj.P.Val** | **GSE230665 status** | **GSE35958 logFC** | **GSE35958 adj.P.Val** | **GSE35958 status** | **GSE7429 logFC** | **GSE7429 adj.P.Val** | **GSE7429 status** | **Cross-cohort class (GSE230665 vs GSE35958)** |
|---|---|---|---|---|---|---|---|---|---|---|
| MAPK1 | -1.09 | 0.0106 | DEG | -1.663 | 0.0301 | DEG | 0.0176 | 0.983 | not DEG | Cross-validated |
| CTNNB1 | -2.08 | 0.0222 | DEG | 2.055 | 0.00678 | DEG | -0.1627 | 0.756 | not DEG | Discordant |
| MMP9 | -1.929 | 0.0113 | DEG | -0.5417 | 0.628 | not DEG | -0.0728 | 0.804 | not DEG | Discovery only |
| VDR | -2.048 | 0.0406 | DEG | 0.9217 | 0.107 | not DEG | 0.1184 | 0.573 | not DEG | Discovery only |
| TNF | 0.2928 | 0.751 | not DEG | -1.917 | 0.0336 | DEG | 0.0266 | 0.929 | not DEG | Validation only |
| MAPK14 | -0.1688 | 0.75 | not DEG | -1.254 | 0.0209 | DEG | 0.1291 | 0.764 | not DEG | Validation only |
| RUNX2 | -1.106 | 0.236 | not DEG | -3.722 | 0.00813 | DEG | 0.0246 | 0.926 | not DEG | Validation only |
| IL6 | -0.4956 | 0.412 | not DEG | 0.0169 | 0.988 | not DEG | 0.003 | 0.995 | not DEG | Non-DEG |
| IL1B | -1.288 | 0.25 | not DEG | -0.7096 | 0.312 | not DEG | 0.112 | 0.71 | not DEG | Non-DEG |
| NFKB1 | -0.8143 | 0.0411 | not DEG | -0.5239 | 0.298 | not DEG | 0.0481 | 0.939 | not DEG | Non-DEG |
| PTGS2 | -2.006 | 0.213 | not DEG | -0.6309 | 0.593 | not DEG | 0.0224 | 0.916 | not DEG | Non-DEG |
| TNFSF11 | -1.086 | 0.344 | not DEG | -0.8388 | 0.63 | not DEG | -0.0287 | 0.907 | not DEG | Non-DEG |
| TRPV1 | -1.438 | 0.0672 | not DEG | | | Absent | | | Absent | Absent |

Cross-validated indicates significant differential expression in both cohorts with the same direction of change; Discordant indicates significant differential expression in both cohorts with opposite directions of change; Discovery only and Validation only indicate significance in a single cohort only; Non-DEG indicates no significant differential expression in either cohort; Absent indicates that the gene was not represented on the corresponding microarray platform.
